# Optimization by the 4S Sequential Experimental Design Process of a Competitive Lateral Flow Immunoassay Device for the Detection of Aflatoxin B1

**DOI:** 10.3390/toxins17110557

**Published:** 2025-11-13

**Authors:** Simone Cavalera, Sofia Stanzani, Thea Serra, Valentina Testa, Fabio Di Nardo, Claudio Baggiani, Laura Anfossi

**Affiliations:** Department of Chemistry, University of Turin, Via Pietro Giuria 7, 10126 Turin, Italy; simone.cavalera@unito.it (S.C.); sofia.stanzani@unito.it (S.S.); thea.serra@unito.it (T.S.); v.testa@unito.it (V.T.); fabio.dinardo@unito.it (F.D.N.); claudio.baggiani@unito.it (C.B.)

**Keywords:** gold nanoparticles, visual detection, multivariate

## Abstract

Aflatoxin B1 (AFB1) is a highly toxic and carcinogenic compound produced by certain fungi (e.g., *Aspergillus flavus* and *Aspergillus parasiticus*). Rapid and ultra-sensitive detection methods for AFB1 in various commodities are in high demand. This study aimed to enhance the sensitivity of a competitive lateral flow immunoassay (LFIA) for AFB1 detection by leveraging a previously developed experimental design strategy, named 4S. This approach comprises four phases—START, SHIFT, SHARPEN, and STOP—and involves the analysis of two reference conditions: NEG (0 ng/mL AFB1) and POS (1 ng/mL AFB1). By generating and overlaying response surfaces, regions of optimal NEG signal and POS/NEG signal ratio (IC%) were identified. Four variables were optimized: two related to the labeled antibody (its concentration and antibody-to-label ratio) and two to the competitor antigen (its concentration and hapten-to-protein ratio). An initial design defined the parameter space, while three subsequent designs did not yield further improvements in sensitivity. A strong anti-correlation was observed between the IC% and competitor parameters. The optimized LFIA-1 exhibited enhanced sensitivity, achieving a limit of detection of 0.027 ng/mL compared to 0.1 ng/mL for the original device. Additionally, the amount of expensive antibody required for device fabrication was reduced by around a factor of four.

## 1. Introduction

Mycotoxins are toxic fungal metabolites that contaminate food and agricultural products, posing a serious risk to human and animal health. In order to limit their harmful effects, many countries have introduced strict regulations [1,2], thereby creating a demand for reliable, sensitive, and cost-effective detection methods. While advanced techniques such as HPLC, LC–MS/MS, and ultra-HPLC offer high accuracy and broad detection capabilities, their high cost, unportability, and the requirement for trained personnel limit their widespread use. On the other hand, immunoassays provide simpler, faster, and more economical alternatives. Among these, lateral flow immunoassays (LFIAs) are particularly appealing as they are portable and have a short measuring time, low cost, high efficiency, and no need for professional operators. However, they face challenges such as environmental sensitivity, material stability, and false results. Therefore, improving the accuracy, reproducibility, and sensitivity of LFIAs is crucial, particularly for highly dangerous mycotoxins such as aflatoxin B1 (AFB1), which is one of the most carcinogenic substances known.

LFIA have become a cornerstone in the field of point-of-care (POC) diagnostics, offering a unique combination of simplicity, portability, and rapid response. These devices are widely employed across a broad spectrum of applications, ranging from clinical diagnostics and veterinary medicine to food safety, environmental monitoring, and biodefense [3,4]. Their operational ease, minimal instrumentation requirements, and capacity for decentralized testing have rendered them particularly valuable in resource-limited settings, aligning well with the World Health Organization’s ASSURED criteria (affordable, sensitive, specific, user-friendly, rapid and robust, equipment-free, and deliverable to end-users) [5].

The application of LFIAs in the detection of mycotoxins has already been recognized as an established detection method that has produced analytical products and matured to the commercialization stage [6,7,8]. While LFIAs have progressed toward commercialization for mycotoxin detection, many reported approaches remain at an early stage and encounter persistent issues including false positives, cross-reactivity, and limited sensitivity. In this context, extensive research has been devoted to achieving high sensitivity in lateral flow immunoassays (LFIAs) for mycotoxins in general, and especially for the detection of aflatoxin B [9,10,11,12,13,14]. Several comprehensive reviews have summarized the recent advances in the field and have critically discussed strategies for achieving the goal of increasing the sensitivity of LFIAs for the detection of aflatoxins [15,16,17,18,19]. These strategies enabled LODs to be reached that are commonly in the range of 0.01–1.0 ng/mL, with some assays achieving ultra-low values down to 0.32 pg/mL. Important parameters have been recognized, such as the use of oriented antibody immobilization, the development of high-affinity recognition reagents and optimized labeling strategies, and signal amplification. However, most of these approaches suffer cost and operational complexity.

Among the various LFIA formats, the colorimetric or visual LFIA is still the most prevalent. This format typically employs gold nanoparticles (AuNPs) as plasmonic reporters that generate a visible signal upon accumulation at the test line. The intense red color of AuNPs, arising from their localized surface plasmon resonance (LSPR), enables naked-eye detection without the need for external instrumentation. This feature, combined with the chemical stability, biocompatibility, and ease of functionalization of AuNPs, has contributed to their widespread adoption in commercial LFIA platforms.

Despite their ubiquity, visual LFIAs are often perceived as low-sensitivity biosensors, especially when compared to fluorescence-based or electrochemical detection systems [20]. However, this belief may be misleading. In many cases, the suboptimal performance of visual LFIAs is not an intrinsic limitation of the detection method, but rather a consequence of insufficient optimization. Indeed, the development of LFIA devices often prioritizes rapid prototyping and manufacturability over rigorous analytical refinement. As a result, many commercial assays operate at levels far from their theoretical performance limits. In recent years, a growing body of research has demonstrated that systematic optimization strategies, particularly those grounded in the design of experiments (DoE) and chemometric approaches, can significantly enhance the analytical performance of LFIA devices. These strategies have been successfully applied to both non-competitive (sandwich-type) and competitive assay formats [21,22,23,24]. While non-competitive LFIAs are generally more straightforward to optimize due to their direct signal–analyte relationship, competitive LFIAs pose unique challenges [23]. In these systems, the signal intensity is inversely proportional to the analyte concentration, complicating both the calibration process and the interpretation of optimization outcomes. The optimization of competitive LFIAs is particularly demanding due to the complex interplay between multiple assay parameters, including the concentration and affinity of the capture and detection reagents, the kinetics of the competitive binding reaction, and the physicochemical properties of the conjugates. Moreover, the decision-making process that guides the optimization—i.e., the selection of the most appropriate response variable or performance metric—is often non-trivial. Traditional metrics such as signal-to-noise ratio or limit of detection may not fully capture the nuances of competitive assay behavior.

**Scheme 1:**
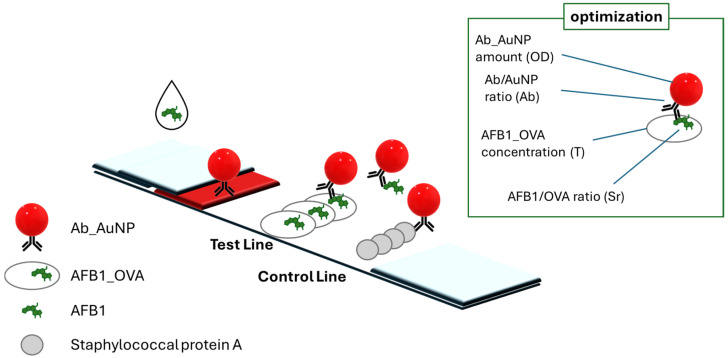
Scheme of the LFIA for measuring AFB1, including: the probe (antibody labeled with AuNP), the competitor (AFB1_OVA) anchored onto the membrane to form the test line, and Staphylococcal Protein A anchored to the membrane to form the control line. The variables included in the optimization of the LFIA test for AFB1 detection were connected with the probe (Ab_AuNP amount and ratio) and with the competitor (AFB1_OVA concentration and ratio).

To address these challenges, we previously proposed a structured optimization framework that we named the 4S method (START, SHIFT, SHARPEN, and STOP). This method integrates experimental design with a decision-tree approach to guide the selection of optimization criteria and parameter ranges [23]. The 4S method was conceived to provide a rational and reproducible pathway for enhancing the sensitivity of competitive LFIAs, particularly in applications where regulatory thresholds demand ultra-low detection limits. In the present study, we apply the 4S method to optimize a LFIA for the detection of AFB1. Schematically (Scheme 1), the LFIA for AFB1 is based on a competitive design in which the target toxin eventually presents in the liquid sample, flows across the LFIA strip, and competes with an AFB1–protein conjugate (competitor) adsorbed on the strip (test line) for the binding to a labeled antibody (detector).

To further expand the efficiency of the 4S strategy in reaching high analytical sensitivity, we introduced an additional variable into the optimization process. In the first application of the method, the variables studied were mainly connected to the characteristics of the detector and the impact of the competitor was investigated by considering only its amount. In this work, we also studied the effect of the substitution ratio (Sr) of the AFB1–protein conjugate immobilized on the test line [25]. The Sr, defined as the average number of hapten molecules covalently attached per carrier protein molecule, can significantly influence the competitive binding dynamics. A low Sr may enhance signal suppression at low analyte concentrations, thereby improving sensitivity, but may also require a higher amount of the competitor to assure the efficient capturing of the probe and an intense signal in the absence of the analyte. Conversely, a high Sr may increase the limit of detection but improve detectability and reproducibility. Despite its potential impact, the Sr is rarely considered as an optimization parameter in LFIA development. In addition, driven by the extremely challenging requirements for sensitivity of AFB1 detection, we considered a new elaboration of DoE results to interpret them, such as the inhibition concentration. This was defined as the ratio between the signal obtained for a sample not containing AFB1 and one containing AFB1 at the concentration chosen as the target for the limit of detection.

The final optimized LFIA was evaluated through performance metrics including the limit of detection (LOD) and the dynamic range. These parameters were then finally compared with the ones obtained in a previous study based on the same antibody [26].

## 2. Results

### 2.1. Variables Associated with the Competitor

The optimization of a competitive LFIA mostly involves parameters associated with the competitor and, in this work, the concentration of the antigen spotted onto the test line (T) and the hapten/antigen ratio were considered. In fact, the competition between the analyte and the competitor for the binding site of the labeled antibody depends not only on the amount of competitor, but also on the characteristics of the competitor itself. This aspect was not explored in our previous work on the 4S optimization because the antigen used as the competitor was a commercial one [26]. In this work, the competitor is an hapten–protein antigen synthesized starting from AFB1 derivatized with O-(Carboxymethyl)hydroxylamine. The resulting oxime (AFB1-CMO) was synthesized and confirmed by mass spectrometry, as previously described [27]. Then, it was coupled with ovalbumin (OVA) by the N-hydroxy succinimide (NHS) and N,N′-diisopropylcarbodiimide (DIC) method [27]. Three molar excesses (10, 40, and 160) were reacted with a fixed number of OVA moles to obtain three different competitors to explore the best compromise between high substitution rates, which improve the affinity with the labeled antibody and generate high signals in the absence of the analyte, and low substitution rates favoring competition with the analyte and, therefore, sensitivity.

**Figure 1:**
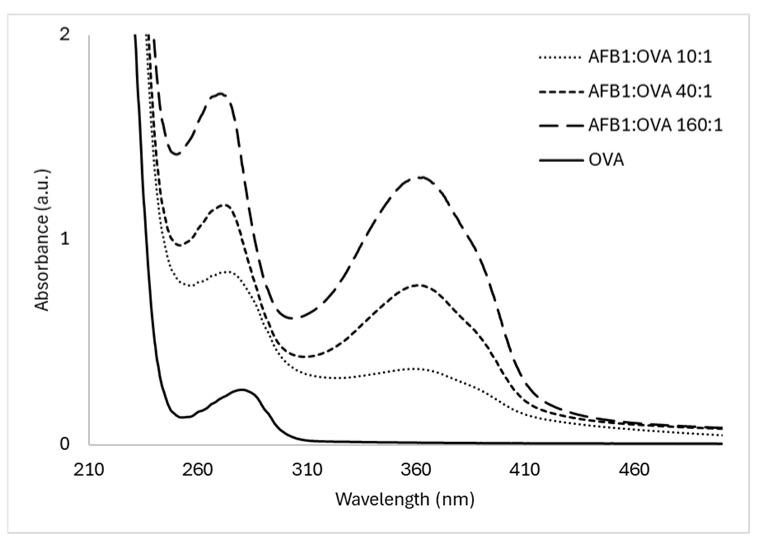
UV–vis spectra of the three AFB1–OVA hapten–protein conjugates prepared and used as the competitors in the DoE.

The resulting three AFB1–OVA conjugates were characterized by UV–vis spectrophotometry (Figure 1). The successful conjugation was confirmed by the presence of the typical band of AFB1 at 365 nm in all conjugates. Moreover, the absorbance increased as a function of the molar excess used in the synthesis, as expected. From spectra, the Sr were estimated as 9.9, 20.7, and 35.1 for the 10-, 40-, and 160-fold molar excesses, respectively.

### 2.2. Preparation of the Gold-Labeled Antibody

**Figure 2:**
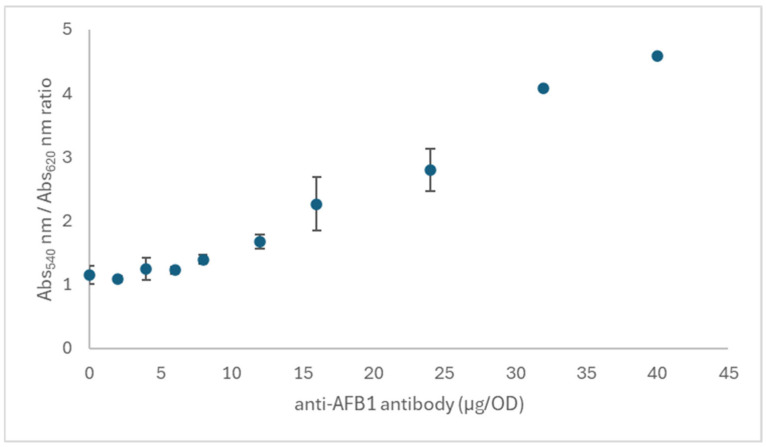
Salt-induced aggregation test for deciding the stabilizing amount of antibody needed to passivate AuNPs with mean diameter of 32 nm at pH 8. The ratio of absorbance at 540 nm (stable colloid) and 620 nm (aggregated AuNP) showed a monotone increase, suggesting that the complete stabilization required higher quantity of the proteins. Bars represent standard deviations of duplicate experiments.

The synthesis of AuNPs was carried out by reduction of gold chloride in the presence of sodium citrate and showed a plasmonic band centered at 525 nm (Figure S1). The gold– antibody conjugates used in the 4S optimization process were obtained by passive absorption of antibodies onto the metallic gold surface. The investigated variables linked to the gold–antibody conjugates were the amount of antibody (Ab) added to 1 mL of AuNP solution characterized by OD 1 at the maximum of the plasmonic band, and the concentration of the gold conjugate in the device (OD). The levels of Ab were defined based on the salt-induced aggregation test as fractions of the minimum stabilizing amount (24 µg), and 0.325, 0.75, 1.5, 3, 6, and 12 µg were used. The assessment of the stabilizing amount was selected as the concentration of Ab providing a ratio between the absorbances at 540 nm and 620 nm higher than 2.5 in the salt-induced aggregation test. The smallest stabilizing amount of Ab was evaluated with the 32 nm AuNPs in a range of 0–40 µg/OD. Compared to other circumstances, where the absorbance ratio reaches a plateau at values of 3–4, in this case an increasing ratio, reaching a value of almost 5, was found with high concentration of Ab (Figure 2). We considered 24 µg/OD as the amount closest to 3 and used this value as the reference point to define the levels of Ab used in the 4S. Considering we were optimizing a competitive test, we must work in defect of Ab, so fractions of the stabilizing amount were used in the DoEs (0.25x = 6 µg/OD, 0.125x = 3 µg/OD, 0.063x = 1.5 µg/OD, 0.031x = 0.75 µg/OD, and 0.016x = 0.375 µg/OD).

The gold–antibody conjugates were characterized by spectrophotometry in the visible range (Figure S1) and the concentration was measured in terms of optical density (OD) in correspondence with the wavelength at the maximum of the localized surface plasmon resonance (LSPR) peak. The desired OD was reached after dilution or concentration (through centrifugation) of the prepared conjugates.

### 2.3. Optimization Process

The levels of AFB1 to test chosen conditions were identified as 0 ng/mL (absence of the analyte, NEG sample) and 1 ng/mL (amount of AFB1 that should be detected by the LFIA, POS sample). In addition, as the LFIA was intended for visual inspection, we decided to apply signal thresholds that corresponded to a clearly visible signal (80 arbitrary units, as measured by the QuantiScan 3.0 (Bio-Rad, Cambridge, UK) software used to process images of the developed device) and a non-perceivable color (15 a.u.).

The starting experimental design of the 4S process (START) included 28 experiments selected by D-optimal among the total of 108 (Figure S2). The results in the absence of AFB1 (NEG) and in the presence of 1 ng/mL of AFB1 (POS) were elaborated by CAT. The multiple linear regression (MLR) elaborated the model reported in Table 1 for the NEG and POS, respectively.

**Figure 3:**
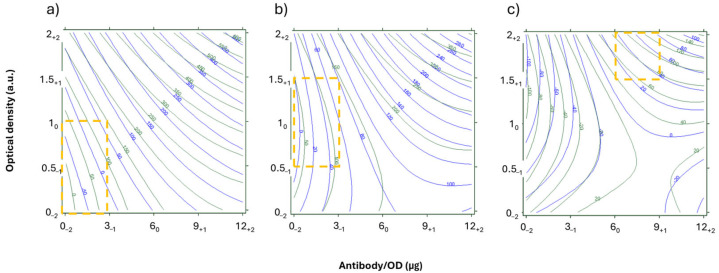
Overlay or response curves for the NEG (green) and POS (blue) samples in the START DoE as a function of the probe parameters. The *x*-axis is antibody/OD ratios, expressed as µg/OD, and *y*-axis is the optical density of the Ab_AuNP, expressed as arbitrary units. The corresponding levels of the DoE are shown as subscripts. The T was fixed at 0.1 mg/mL and Sr were 35.1 (**a**), 20.7 (**b**), and 9.9 (**c**). Yellow squares showed regions where NEG were above 80 a.u (true negative) and POS were below 15 a.u. (true positive).

The analysis of the significance of the coefficients showed that, in both the datasets, the four variables were all significantly and positively correlated in the explored parameter space (Figure S3). This confirms that the levels and the variables were carefully chosen but makes the identification of a “key variable” for the competition quite difficult. In fact, the same improvement will increase both the signal due to the binding between the labeled antibody and the competitor spotted onto the test line, but also the residual “not-inhibited signal.” By overlaying the NEG and POS response surfaces, this aspect is visually clear (Figure S4), as shown in the example in Figure 3. Extremely limited areas satisfy the criteria (NEG > 80 a.u. + POS < 15 a.u.). In addition, they lie close to the limit for the Ab variable (0 µg). This is, probably, because the system sensitivity is limited for 1 ng/mL of AFB1. Therefore, another result was plotted in order to reveal the potential sensitivity without other experiments.

To this purpose, we used the inhibition capacity (IC%) in place of the POS results. The IC% was calculated as the (NEG-POS)/NEG × 100.

The dataset was processed similarly to the POS, the resulting model was obtained, and the coefficient plot showed that pair combinations’ terms coefficients were not significant (Figure S5), and the dataset was re-elaborated excluding these terms (Table 1).

**Figure 4:**
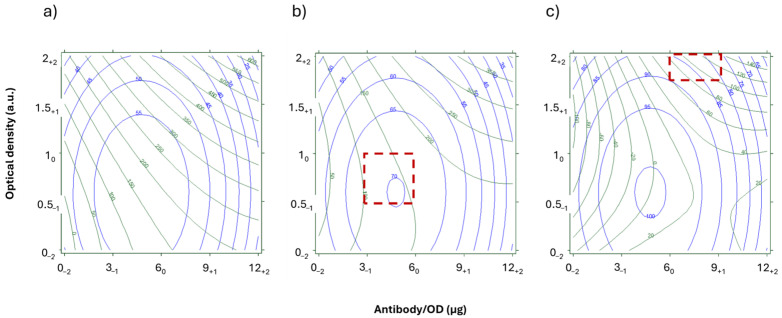
Overlay or response curves for the NEG (green) and IC% (blue) samples in the START DoE. The T was fixed at 0.1 mg/mL and Sr were 35.1 (**a**), 20.7 (**b**), and 9.9 (**c**). The *x*-axis represents antibody/OD ratios, expressed as µg/OD, and *y*-axis represents the optical density of the Ab_AuNP, expressed as arbitrary units. The corresponding levels of the DoE are indicated as subscripts. Red squares show regions where NEG were above 80 a.u (true negative) and POS were below 15 a.u. (true positive).

Contrarily to the elaboration of the POS dataset, the IC% coefficient plot individuated the Sr as an important key variable for the competition since it was highly negatively correlated to the IC% (high significance). T has also shown a significant but weak negative correlation with IC%. The response surfaces of the NEG were overlayed onto the IC% (Figure S6), highlighted the appearance of the high inhibition capacity, ranging from 70% to 85%, associated with the lowering of the Sr of the competitor, as shown in Figure 4. This is consistent with the theory of immunoassay, in that high competition is given by using defect of antibody and competing antigen. It seems that in a lateral flow immunoassay format, where the system is not at equilibrium and there are not washing steps, the hapten amount of the conjugate impacts much more than the defect of antibody, either in terms of Ab or OD. The best combination of variables that can be extracted from this DoE was selected as LFIA-1, characterized by Ab = 6 µg, OD = 2, T = 0.1 mg/mL, and Sr = 9.9.

To further explore the parameter space, a zoom in the interval suggested by the overlay between the NEG and the POS data was designed, including the following ranges: Ab = 0.75–1.5–3 µg/OD, OD = 0.25–0.5–1, T = 0.2–0.4–0.6 mg/mL, and Sr = 20.7–35.1 (SHARPEN 1). The number of experiments was lowered from 54 to 29 by D-optimal.

Another DoE was performed following the suggestion from the overlay between the NEG and the IC% data, including the following ranges: Ab = 0.75–1.5–3 µg/OD, OD = 1–2–3, T = 0.2–0.4–0.6 mg/mL, and Sr = 9.9 (SHARPEN 2). The number of experiments was lowered from 27 to 14 by D-optimal.

One of these routes is an alternative to the other, depending on the clarity of the indications given by the way the first DoE was processed (POS or IC%). In this work we will follow both of them to understand whether the results would be similar at the end of the 4S process.

The data from the SHARPEN 1 experimental design were processed both as NEG/POS and NEG/IC% comparisons (Table S1). The model from the NEG includes all the variables, while in the model for the POS the nonsignificant variables (square and pair combination terms) were excluded basing on the significance of the coefficient shown in the coefficient plots (Figure S7 and Table S2). Also, the pair combinations were excluded from the model of the IC%**.** The models obtained with NEG, POS, and IC% datasets were obtained (Table S3). The NEG/POS (Figure S8) overlay was obtained and areas satisfying the criteria were found in all T and Sr conditions at Ab = 6 µg/OD and OD = 0.25 but the experiments performed with these conditions did not confirm this prediction, showing low IC% and high values for POS experiments. Also, the NEG/IC% (Figure S9) overlays were obtained; however, no zones were found having >50% of IC%, which was consistent with the results of the verification experiments. The coefficients plot of the IC% highlighted the significative moderate negative correlation for the Sr, and weakly significative negative correlation for the T. Despite this information, considering the absence of an improvement in the space, no further experiments were performed in this direction.

**Figure 5:**
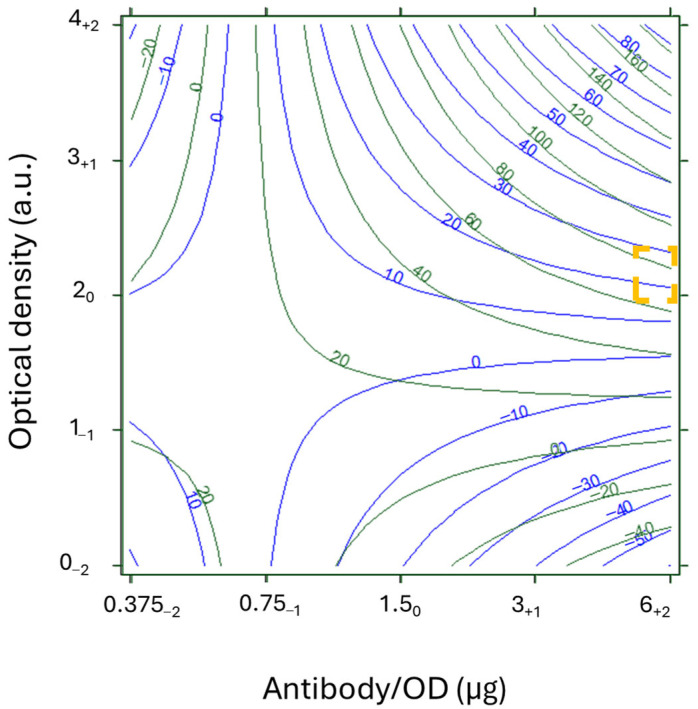
Overlay of response curves for NEG (green) and POS (blue) in the SHARPEN 2 DoE, detail for T = 0.2 mg/mL and Sr = 9.9. The *x*-axis represents antibody/OD ratios, expressed as µg/OD, and *y*-axis represents the optical density of the Ab_AuNP, expressed as arbitrary units. The corresponding levels of the DoE are indicated as subscripts. The yellow square identifies a region where NEG was above 80 a.u (true negative) and POS was below 15 a.u. (true positive).

Concerning the SHARPEN 2 experimental design, the models were extracted, and the coefficients’ plots were analyzed to individuate the significant variables, to re-elaborate the models without nonsignificant terms (Figure S10). The following models were used for the NEG (without square terms), POS, and IC%, respectively:

The NEG/POS overlay from SHARPEN 2 (Figure S11) highlighted, essentially, the same experimental zone already found by the NEG/IC% plot from the START design (Figure 5).

**Figure 6:**
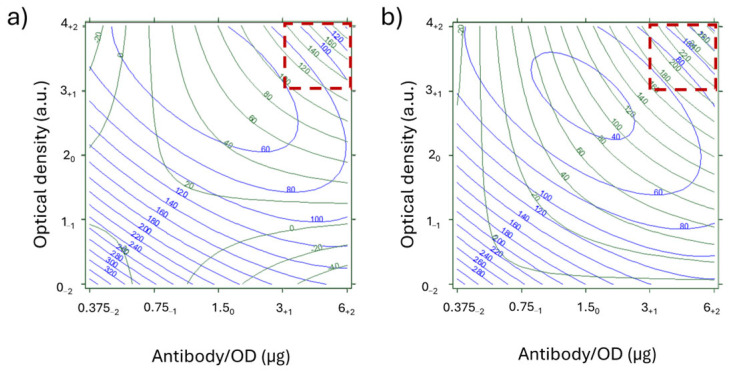
Overlay of response curves for NEG (green) and IC% (blue) in the SHARPEN 2 DoE. Sr was fixed at 9.9 and T values were 0.2 mg/mL (**a**) and 0.4 mg/mL (**b**). The *x*-axis represents antibody/OD ratios, expressed as µg/OD, and *y*-axis represents the optical density of the Ab_AuNP, expressed as arbitrary units. The corresponding levels of the DoE are indicated as subscripts. Red squares show regions where NEG were above 80 a.u (true negative) and POS were below 15 a.u. (true positive).

The IC% coefficient plot showed, in the absence of the variable Sr, high negative correlation, especially with OD, and moderate negative correlation with Ab and T. The NEG/IC% overlay (Figure S12), on the other hand, showed a different area where the predicted IC% was above 80% (Figure 6). This area was further explored in the SHARPEN 3 experimental design, which included an Ab between 3 and 6 µg/OD, optical densities between 3 and 4, and concentrations on the test line between 0.2 and 0.4 mg/mL, while the Sr was kept constant at 9.9. The results were processed (Table S5), as described above, and the coefficients’ plots were used to re-elaborate the models excluding nonsignificant coefficients (Table S1). No significative variables were found in the explored interval (Figure S13). Observing the NEG/POS (Figure S14) overlay, high values of POS were the reason why it was not possible to overcome the performances of the LFIA-1 device. Looking at the NEG/IC%, it is clear that no conditions provided an IC% above 50% (Figure S15), and the parameters identified as the LFIA-1 remained optimal.

### 2.4. Analytical Evaluation of the Optimized LFIA-1 for AFB1 Detection

**Figure 7:**
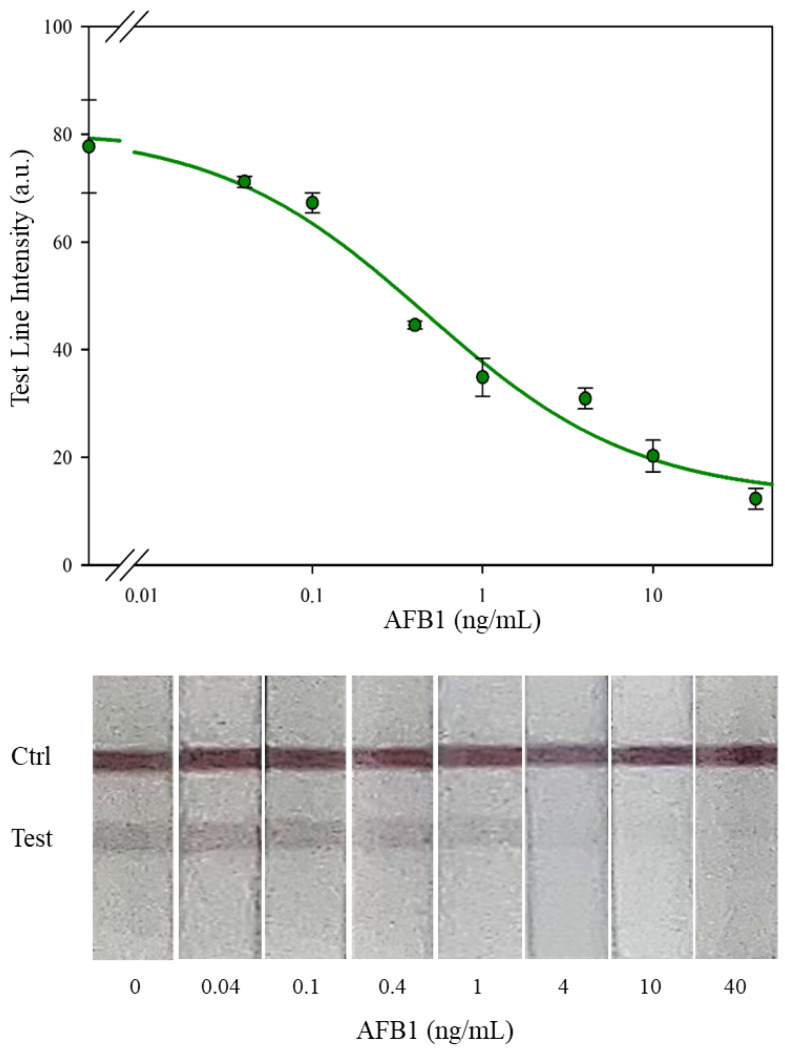
Calibration curve for the LFIA-1: the test line intensity was plotted towards AFB1 concentration and fitted by the 4-PL equation (green line). Bars represent standard deviation of duplicate experiments. In the bottom, examples of pictures of the analytical windows of the LFIA devices.

Calibration curves for AFB1 diluted in buffer were made with the LFIA-1 (Figure 7) and the results were compared with a previous work in the literature describing the development of a LFIA for AFB1 detection using the same antibody and a competing antigen made with the same hapten (AFB1-cmo) [26] (Table 2).

When comparing them, the amount of antibody and competitors are in the same order of magnitude. In detail, LFIA-1 included the same OD of labeled antibody, with a lower Ab/AuNP ratio (6 vs. 10 µg/OD) and half of the competitor (0.1 vs. 0.2 mg/mL) as the pristine device. To reach the optimal condition, the 4S needed 56 experiments (28 conditions tested for 2 AFB1 concentrations, 0 and 1 ng/mL) while the separate determination of the three observed variables in 2011 required 11 conditions to be explored by building the whole calibration curve (7 levels), for a total of 77 experiments. The instrumental sensitivity parameters of the LFIA-1 were all superior to the unoptimized values: IC_50_ 0.7 (vs. 2.4) ng/mL, dynamic range 0.09–13 (vs. 0.2–4) ng/mL, and LOD 0.027 (vs. 0.1) ng/mL. All these parameters were calculated on a T/C ratio curve just to compare with the data of the unoptimized LFIA. The improved sensitivity reflected in the qualitative output of the optimized device (contrary to what happened in the unoptimized LFIA), the LFIA-1, showed the complete disappearance of the test line at 10 ng/mL AFB1, which was then considered as the visual limit of detection (vLOD) of the method.

**Figure 8:**
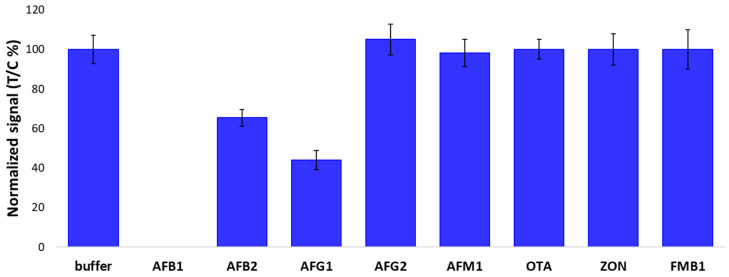
Selectivity of the optimized LFIA towards major aflatoxins and mycotoxins. The signal measured at the test line was divided by the signal measured at the control line (T/C) and normalized by the T/C value calculated for the buffer that did not contain any AFB1.

The selectivity of the optimized LFIA was evaluated by analyzing other regulated aflatoxins (AFG1, AFB2, AFG2, and AFM1) and mycotoxins (ochratoxin A, zearalenone, and fumonisin B1), each one at 10 ng/mL corresponding to the vLOD of the device in the presence of AFB1. As expected, based on the cross-reactivity pattern of the antibody used, other aflatoxins (AFs) showed measurable inhibition of the blank signal, in the following order: AFB1 > AFG1 > AFB2 > AFG2. Mycotoxins with chemical structures that are not similar to that of AFB1 were not recognized by the LFIA (Figure 8).

At the same time, the robustness towards matrix effects was studied by fortifying two flour samples (maize and wheat), which are representative of real samples that are typically prone to AFB1 contamination. The samples were confirmed to contain AFB1 below the limit of detection of the LFIA by HPLC-MS analysis and were analyzed before and after fortification with a known amount of toxin. The matrix effect was confirmed to be negligible for both samples: the blanks provided signals comparable to those obtained for the calibrator ‘0’, and the addition of low (0.1 ng/mL), medium (1 ng/mL), and high (10 ng/mL) levels of AFB1 provided the progressive inhibition of the signal, as observed for the buffered solutions (Table 3).

## 3. Discussion

Comparing this work with the previous work involving the 4S optimization process, two aspects are evident. The first is the fact that a lower number of experiments was necessary for finding an improved sensitivity conditions combination. Nevertheless, to gain an understanding of the highest sensitivity, many experiments were performed to conclude that no improvement was possible beyond the LFIA-1. The use of the IC% as a figure of merit, combined with the overlapping of NEG results, helped in this process, rationalizing the results of the inhibition in the absence of TP + TN areas. The second important aspect is the addition of the Sr as a variable, which was extremely impactful. Clearly, in the scenario of the optimization of a competitive LFIA, the Sr is not always a variable that can be chosen. But this work evidenced that, similarly to what happens in the ELISA platform, the Sr of the competitor has a critical impact on sensitivity.

From Table 2 it is evident that the LFIA-1 provided better analytical performances compared with the one from Anfossi et al. in 2011 [26], although the two works were not completely comparable. In fact, in the pristine work the competitor was a bioconjugate formed by BSA instead of OVA and the molar excess was 200:1. Another important difference is given by the T/C ratio used for the evaluation of analytical performances in the previous work, which required a reader and the processing of the signals. In that case the control line was also calibrated to lower much more in the absence of analyte because the labeled antibody was fully captured by the test line and no excess was provided to the control line. This escamotage allowed for the improvement of the instrumental sensitivity but is completely useless for qualitative analysis, where the control line must always be present and the test line must disappear in the presence of a certain concentration of the analyte.

Despite of these differences, both devices relied on the same antibody and the same hapten. This fact is crucial to honestly evaluate the added value given by the 4S optimization procedure itself. (Almost) the same reagents were combined without (Anfossi et al., 2011 [26]) and with (present work) the 4S approach, and the result is its superiority in terms of results and reagent consumption.

Notably, the optimization process did not alter the cross-reactivity pattern of the system, which gave the expected results, based on the cross-reactivity pattern of the antibody used.

The cross-reactivity, in fact, followed the chemical similarity with the other aflatoxins (AFB1 > AFG1 > AFB2 > AFG2) and there was no cross-reactivity for those that were completely different (OTA, ZON, FMB1).

What was very important, and less obvious, was the fact that the matrix effect also remained under control. In fact, the optimized gold conjugates could have showed less stability or proneness to matrix interference, maybe due to less coverage of the AuNPs with the antibody, which is also the stabilizing agent, to improve the sensitivity favoring the competition. The overcoating step in the conjugation, as well as the additives in the buffer, seem to be sufficient to protect the gold conjugate.

The 4S approach is an efficient route to optimize the performance of the common and widely used LFIA method, and can also be compared to the few previous works reporting the performance of lateral flow immunoassays based on using antibodies as the recognition element, gold nanoparticles as the label, and visual/colorimetric detection (Table 4).

All the studies cited, except for that of Anfossi et al. [26], were conducted using the same materials and format (visual detection and gold nanoparticles as the label) but different bioreagents (antibody and antigen), so the comparison is not valid in terms of evaluating the optimization method, which is the focus of this study.

## 4. Conclusions

Key parameters from the previous cortisol LFIA optimization were also relevant for AFB1 detection. The newly investigated parameter Sr significantly influenced assay sensitivity. Through 28 experiments (START DoE), the 4S approach improved signal inhibition from 0% to 70–85% at 1 ng/mL AFB1. While POS signal criteria did not yield suitable conditions for SHARPEN 1, using IC% as the metric enabled identification of optimized LFIA-1 conditions. A second SHARPEN 2 design (14 experiments) based on NEG/IC% overlays revealed another optimization zone. The final LFIA achieved an LOD of 0.027 ng/mL, outperforming conventional gold nanoparticle-based methods.

In fact, most of the recent literature regarding the development and optimization of LFIA for AFB1 detection reported new probes, detected signals, signal enhancement strategies, or the use of recognition elements other than antibodies [15,16,17,18,19]. This demonstrates that applying the 4S strategy with selected immunoreagents can enhance sensitivity with fewer experiments and reduced reagent costs. It should be noted that the drawback is the fact that some experiments performed for further improving the sensitivity were useless apart from being a confirmation of the results from the START DoE. A simple and inexpensive strategy would be needed to understand when maximum sensitivity has been reached.

## 5. Materials and Methods

### 5.1. Synthesis of the AuNPs

The synthesis of the AuNPs was carried out using sodium citrate reduction of a boiling aqueous solution of chloroauric acid, as previously reported [33]. The procedure is detailed in the Supplementary Information (SI).

### 5.2. Salt-Induced Aggregation Test

To determine the minimum stabilizing concentration of Ab for 32 nm AuNPs, the salt-induced aggregation test was performed, as previously described [23] and detailed in the Supplementary Information (SI). The stabilizing amount was defined as the Ab concentration that produced a ratio of absorbencies at 540 and 620 nm greater than 2.5.

### 5.3. Synthesis of Gold Conjugates Ab_AuNPs

The synthesis of the gold conjugates used in the 4S optimization process was carried out as previously described [23]. Briefly, 1 mL of AuNPs with an optical density of ca. 1 was centrifuged at 12,000× *g* for 12 min. The supernatant was then removed and replaced with 10 mM Tris-HCl buffer at pH 8.5, and the pellet was resuspended by sonication.

The desired amount of antibody was determined based on the salt-induced aggregation test. During the 4S process, 0.325, 0.75, 1.5, 3, 6, and 12 µg were added to the AuNPs, which were then incubated at 37 °C for 30 min, followed by the addition of 100 µL of borate buffer (20 mM, pH 8) supplemented with 1% BSA, and further incubation for 10 min. The gold conjugate was then purified by three centrifugation cycles of 12 min each at 25 °C and 15,000× *g*.

After the first centrifugation, the supernatant was replaced with a 0.1% *w/v* BSA solution in borate buffer. After the second centrifugation, the supernatant was replaced with a storage buffer solution containing 1% *w/v* BSA, 2% *w/v* sucrose, 0.025% *v/v* Tween 20, and 0.02% *w/v* NaN_3_ in borate buffer (20 mM, pH 8). After the third centrifugation, the pellet was reduced to approximately one-twentieth of its original volume by removing the supernatant. Finally, the pellets obtained from each mL of synthesis were pooled and mixed. A small aliquot was diluted 40-fold and used to measure the optical density of the gold conjugate by UV–vis spectroscopy on a Cary 60 UV-vis spectrometer (Agilent, Santa Clara, CA, USA).

### 5.4. Synthesis of the Antigens (AFB1_OVA)

The competing antigens used as the capture agent were synthesized using a well-established protocol. This involved the formation of an amide bond between carboxylic groups and amines after the conversion of the carboxyl into an N-hydroxy succinic ester in the presence of N-hydroxy succinimide (NHS) and N, N′-diisopropylcarbodiimide (DIC). In this case, the carboxyl carrier is a hapten consisting of aflatoxin B1 and a spacer arm (AFB1-CMO), which was synthesized as previously described [27]. Specifically, the AFB1-CMO was dissolved in anhydrous DMF and incubated for 1 h with 1.1 equivalents of DIC and 1.1 equivalents of NHS. The mixture was then added to a freshly prepared solution of ovalbumin (OVA) in sodium hydrogen carbonate buffer (150 mM, pH 8.5) at various ratios to produced different competitors. The reactions stoichiometric ratios (RM_0_) were 10, 40, and 160. After overnight reaction, the purification was carried out by using Sephadex G25 cartridges (GE Healthcare, Little Chalfont, UK). The fractions corresponding to the AFB1-OVA products were analyzed to determine the substitution ratio (Sr). Uv–vis spectra were obtained in the range 200–450 nm using a Cary 60 (Agilent, Santa Clara, CA, USA) spectrophotometer and the concentration of AFB1 was estimated from the absorbance at 365 nm by using an extinction coefficient of 3411 M^−1^ cm^−1^, which was calculated from calibration curve made with 0–0.5–1.0–1.5–2.0–2.5 × 10^−4^ M solution of AFB1 in phosphate buffer pH 7.4 + 0.13 M NaCl. OVA was quantified by Bradford’s method. The Sr was calculated as the ratio of the molar concentrations of the AFB1 haptens to the molar concentration of the protein.

### 5.5. Execution of the LFIA and Acquisition of the Analytical Response

The LFIA devices were fabricated as previously reported [23], with the minor modifications detailed in the Supplementary Information (SI).

Standard solutions of AFB1 diluted in the running buffer were used for the development and optimization of the LFIA. The running buffer formulation consisted of a phosphate buffer at a pH of 7.4, supplemented with 1% *w/v* BSA and 0.1% *v/v* Tween 20. Assay execution consisted of adding 80 µL of the standard solution to the sample well and evaluating the results after 15 min of capillary flow at room temperature. The color intensity of the test lines was visually inspected and digitally acquired using a scanner (OpticSlim 550 scanner, Plustek Technology GmbH, Norderstedt, Germany). QuantiScan 3.0 software (Bio-Rad, Cambridge, UK) was used to analyze the area including the colored lines, and the area of the peak at the test and control lines was used as the “color intensity”, expressed in arbitrary units.

### 5.6. The 4S Optimization of the LFIA for AFB1 Detection

The following variables were investigated simultaneously in the DoE: the amount of anti-AFB1 antibody (Ab) adsorbed onto AuNP, the amount of gold conjugate included in the assay (measured as the optical density (OD) of the Ab-AuNP solution), the concentration of AFB1-OVA spotted to form the test line (T), and its substitution rate (Sr). The number of experiments was defined using the D-optimal algorithm, as previously described [21,22,23,24].

The START design included four levels of Ab (3, 6and 12 µg/OD), three levels of OD (0.5, 1, and 2), four levels of T (0.05, 0.1, 0.2, and 0.4 mg/mL), and three estimated substitution rates (Sr: 9.9, 20.7, and 35.1). The full factorial yielded 108 experiments, which was reduced to 28 by D-optimal (Table 5).

The SHARPEN 1 design included three levels of Ab (0.75, 1.5, and 3 µg/OD), three levels of OD (0.25, 0.5, and 1), three levels of T (0.2, 0.4, and 0.6 mg/mL), and two levels of Sr (20.7 and 35.1). There were 54 full factorial experiments, which were reduced to 28 using D-optimal.

The SHARPEN 2 design included three levels of Ab (0.75, 1.5, and 3 µg/OD), three levels of OD (1, 2, and 3), two levels of T (0.2, 0.4, and 0.6 mg/mL), and one level of Sr (9.9), with 18 full factorial experiments. This was reduced to 14 experiments using D-optimal.

The SHARPEN 3 design included three levels of Ab (3–6 µg/OD), three levels of OD (3–4), two levels of T (0.2–0.4 mg/mL), and Sr = 9.9, with eight full factorial experiments.

In the various DoEs, the levels were centered on the median value and coded using a simple function (linear or exponential) that linked the codes to the variable values.

All conditions were evaluated three times: once in the presence of a defined concentration of AFB1 (POS) and twice in the absence of analyte (NEG). The AFB1 concentration in the POS experiments was 1.0 ng/mL. Additionally, the IC% response surfaces, calculated as (NEG − POS)/NEG × 100, were used in the optimization process as an alternative to the POS signal.

The Chemometric Agile Tool software [34], freely available online within the R version 3.1.2, was employed for multiple linear regression analysis (MLR) model computation and visualization (coefficient plots and response surfaces). Initially, the regression coefficients of the model describing the effect of the variables (Ab, OD, and T) on the response (R, or color intensity of the test lines) were computed considering the constant term (a0), the linear effects of the three variables (a1 to a4), the pair combinations of the variables (a5 to a10), and the quadratic terms of the variables (a11 to a14).

R = a_0_ + a_1_Ab + a_2_OD + a_3_T + a_4_Sr + a_5_Ab × OD + a_6_Ab × T + a_7_Ab × Sr + a_8_OD × T + a_9_OD × Sr + a_10_T × Sr + a_11_Ab^2^ + a_12_OD^2^ + a_13_T^2^ + a_14_Sr^2^

The *p*-values of the regression coefficients were evaluated to identify the statistically significant parameters and visualize the relationships among the parameters involved in the design of experiments (DoE). 

After this evaluation, if no significance was found in an entire effect of coefficients (e.g., all pair combinations or all quadratic terms), the coefficients were excluded from the model, which was then re-elaborated considering only the significant effects.

Experiments under different conditions were performed randomly to reduce the impact of potential systematic error on the calculated model.

### 5.7. LFIA-1 for AFB1 Detection

Calibration curves were obtained by analyzing standard AFB1 solutions in the range of 0–40 ng/mL using the optimized LFIA. The LFIA 0 condition was the starting point for developing the device, during which the immunoreagents were screened for suitability in the expected format, and the main parameters were not optimized. The amount of antibody adsorbed onto AuNPs was 6 µg/OD; the probe concentration was OD = 2; the antigen concentration was 0.1 mg/mL; and the hapten–protein antigen substitution rate was 9.9.

Each calibrator was measured in triplicate. The calibration curves were fitted using four-parameter logistic curves with SigmaPlot 14.0 (Systat Software, Inc., Palo Alto, CA, USA) and the following parameters were calculated: color intensity in the absence of cortisol (A_(max)), half-maximal inhibition capacity (IC_50_), dynamic range (IC_20_–IC_80_), and visual limit of detection (vLOD). The vLOD was calculated by substituting 15 a.u. into the 4PL model. We also evaluated these parameters by using the ratio between the test and control lines (T/C) to compare the sensitivity of the optimized test with that of previous tests prepared with the same materials that were similarly evaluated [18].

The analytical performance was calculated as follows: the LOD was the concentration of AFB1 providing 10% inhibition of the T/C ratio, or the ratio of the color of the test line (T) to the color of the control line (C). The IC_50_ was calculated from the four-parameters logistic curve describing the inhibition of the T/C versus the AFB1 concentration. The IC_50_ is the concentration providing 50% inhibition. The visual LOD (vLOD) was calculated as the AFB1 concentration at which the test line disappeared.

Selectivity was calculated by testing the optimized LFIA in the presence of other regulated aflatoxins (AFG1, AFB2, AFG2, and AFM1) and mycotoxins (ochratoxin A, zearalenone, and fumonisin B1); each one was at 10 ng/mL corresponding to the vLOD of the device in the presence of AFB1. The signal measured at the test line was divided by the signal measured at the control line (T/C) and normalized by the T/C value calculated for the buffer that did not contain any AFB1.

The matrix effect was evaluated as follows: blank samples were extracted and fortified with AFB1 at low, medium, and high levels of the toxin (0,1–1 and 10 ng/mL, respectively) and analyzed in duplicate in parallel to the standard solutions prepared in buffer. The percentage inhibition was calculated as the color intensity measured at the test line (T) divided by the color intensity measured in the absence of AFB1 (T0).

## Figures and Tables

**Table 1:** Coefficients of the model elaborated by CAT after the multivariate linear regression from the START design of experiments. Values were obtained by analyzing a blank solution (NEG) and a solution containing 1 ng/mL of AFB1 (POS). The IC% was calculated as (NEG-POS)/NEG × 100.

	NEG	POS	IC%
b_0_	248.049	159.016	61.376
x Ab	62.284	64.150	−3.967
x OD	57.189	48.063	−2.939
x T	79.793	60.611	−11.109
x Sr	133.961	109.489	−21.981
x AbxOD	13.600	14.441	n.c. ^a^
x AbxT	11.316	19.279	n.c. ^a^
x AbxSr	16.797	20.835	n.c. ^a^
x ODxT	22.622	17.666	n.c. ^a^
x ODxSr	22.003	20.158	n.c. ^a^
x TxSr	13.295	19.383	n.c. ^a^
x Ab^2^	−11.142	−7.690	−4.836
x OD^2^	11.119	6.509	−1.831
x T^2^	8.596	12.421	−4.297
x Sr^2^	−41.849	−30.959	8.356
EV% ^b^	95.88	95.61	85.55

^a^ n.c.: not considered in the model and ^b^ explained variance.

**Table 2:** LFIA parameters optimized in this work (LFIA-1) compared to those defined in a previous work employing the same antibody.

	LFIA-1	Anfossi et al., 2011 [26]
Ab (µg/OD)	6	10
OD (a.u.)	2	2
Sr (ratio)	9.9	n.p. ^f^
T (mg/mL)	0.1	0.2 ^g^
# of exp ^a^ (× points)	28 (×2)	11 (×7)
NEG (a.u.)	81	n.p. ^f^
IC_50_ ^b^ (ng/mL)	0.7	2.4
dynamic range ^c^ (ng/mL)	0.09–13	0.2–4
LOD ^d^ (ng/mL)	0.027	0.1
vLOD ^e^ (ng/mL)	10	n.o. ^f^

^a^ Number of experiments needed to reach the optimized device. ^b^ Calculated from the 4PL curve as the concentration providing the 50% of inhibition of the color intensity of the T/C ratio, where T is the color intensity on the test line e and C is the color intensity on the control line. ^c^ The range of AFB1 concentration comprised between those providing 20% and 80% of the T/C ratio. ^d^ The limit of detection calculated as the concentration providing the T/C ratio higher than the blank + 3 standard deviations. ^e^ Visual LOD corresponding to the concentration providing the disappearance of the test line. ^f^ Not provided in the article. ^g^ A different competitor was used in Anfossi et al. [26]. (a commercial conjugate between AFB1 and Bovine Serum Albumin, BSA).

**Table 3:** Matrix effect of the optimized LFIA due to maize and wheat flour. Blank samples were extracted and fortified with AFB1 at low, medium, and high levels of the toxin (0.1–1 and 10 ng/mL, respectively) and analyzed in duplicate in parallel to the standard solutions prepared in buffer. The percentage inhibition was calculated as the color intensity measured at the test line (T) divided by the color intensity measured in the absence of AFB1 (T_0_).

Sample	AFB1 Added (ng/mL)	Test Line ± SD ^a^ (a.u.)			Inhibition (%)
buffer	0	83	±	3	100
	0.1	68	±	2	82
	1.0	35	±	4	42
	10.0	0	±	0	0
Maize flour	0	87	±	2	100
	0.1	73	±	3	84
	1.0	34	±	1	39
	10.0	0	±	0	0
wheat flour	0	90	±	2	100
	0.1	72	±	5	80
	1.0	36	±	4	40
	10.0	0	±	0	0

^a^ *n* = 2.

**Table 4:** Comparison of the limits of detection achieved in this work compared to those of colorimetric LFIA using gold nanoparticles and reported in works published in the last ten years (2016–2025).

Year of Publication	Limit of Detection	Ref.
2016	0.1–0.13 µg/kg in corn, rice, and peanuts	[28]
2016	1.5 µg/kg in oil	[29]
2019	0.5 µg/kg in potable water	[30]
2022	0.23 ng/mL	[11]
2025	0.049 ng/mL ^a^	[31]
2025	0.2 ng/mL ^b^	[32]
2025	0.027 ng/mL	This work

^a^ The antibody used was cross-reactive to the group of aflatoxins. ^b^ The LOD for the colorimetric LFIA was reported as the benchmark for comparing the improvement obtained in the work through the employment of a deep-learning assisted detection strategy.

**Table 5:** DoE codification of levels and corresponding values.

	Ab		OD		T		Sr	
DoE	Code	(µg/OD)	Code	(a.u.)	Code	(mg/mL)	Code	Ratio
START	2	12	2	2	1	0.4	2	35.1
	0	6	0	1	0	0.2	0	20.7
	−1	3	−1	0.5	−1	0.1	−1	9.9
					−2	0.05		
SHARPEN 1	1	3	1	1	1	0.6	1	39
	0	1.5	0	0.5	0	0.4	−1	23
	−1	0.75	−1	0.25	−1	0.2		
SHARPEN 2	1	3	1	3	1	0.6		
	0	1.5	0	2	0	0.4		
	−1	0.75	−1	1	−1	0.2		
SHARPEN 3	1	6	1	4	1	0.4		
	−1	3	−1	3	−1	0.2		

## Data Availability

The original contributions presented in this study are included in the article/Supplementary Material. Further inquiries can be directed to the corresponding author(s).

## Supplementary Materials

The following supporting information can be downloaded at https://www.mdpi.com/article/10.3390/toxins17110557/s1: Figure S1: Visible spectra of the gold conjugates; Figure S2: D-optimal evaluation of the number of experiments; Figure S3: The coefficient plot of the NEG and POS models form the datasets; Figure S4: The overlay between NEG and POS models from the START datasets; Figure S5: The coefficient plot of the IC% from the START datasets; Figure S6: The overlay between NEG and IC% models from the START datasets; Figure S7: The coefficient plot of the NEG, POS, and IC% from the SHARPEN 1 dataset; Figure S8: The overlay between NEG and POS models from the START datasets; Figure S9: The overlay between NEG and IC% models from the START datasets; Figure S10: The coefficient plot of the NEG, POS, and IC% from the SHARPEN 2 datasets; Figure S11: The overlay between NEG and POS models from the SHARPEN 2 datasets; Figure S12: The overlay between NEG and IC% models from the SHARPEN 2 datasets; Figure S13: The coefficient plot of the NEG, POS, and IC% from the SHARPEN 3 datasets; Figure S14: The overlay between NEG and POS models from the SHARPEN 3 datasets; Figure S15: The overlay between NEG and IC% models from the SHARPEN 3 datasets; Table S1: the coefficients of the multivariate model obtained from the START, SHARPEN 1, SHARPEN2, SHARPEN 3 datasets including the significant classes of coefficients; Table S2: START dataset; Table S3: SHARPEN 1 dataset; Table S4: SHARPEN 2 dataset; and Table S5: SHARPEN 3 dataset.
