# Supplementary material for: Optimization by the 4S Sequential Experimental Design Process of a Competitive Lateral Flow Immunoassay Device for the Detection of Aflatoxin B1
Source: Toxins (Basel). 2025 Nov 13;17(11):557. doi: 10.3390/toxins17110557 (PMC12656006; doi:10.3390/toxins17110557)
Supplement: Supplementary file 1 [file toxins-17-00557-s001.zip › toxins-3933601-supplementary.pdf]

Article

Optimization by the 4S Sequential Experimental Design Process of a Competitive Lateral Flow Immunoassay Device for the Detection of Aflatoxin B1

Simone Cavalera, Sofia Stanzani, Thea Serra, Valentina Testa, Fabio Di Nardo, Claudio Baggiani and Laura Anfossi *

Department of Chemistry, University of Turin, Via Pietro Giuria 7, 10126 Turin, Italy; simone.cavalera@unito.it (S.C.); sofia.stanzani@unito.it (S.S.); thea.serra@unito.it (T.S.); v.testa@unito.it (V.T.); fabio.dinardo@unito.it (F.D.N.); claudio.baggiani@unito.it (C.B.)

* Correspondence: laura.anfossi@unito.it

SUPPLEMEMTARY INFORMATION

Synthesis of the AuNPs

A total of 10 mg of $AuCl_4$*H_2O was dissolved in 1 mL of milliQ water in a dark glass vial and then transferred and diluted in a volumetric flask at a final concentration of 0.01 % w/v. The solution was heated up to 100 °C and the appropriate amount of sodium citrate was added from a 1 % w/v aqueous solution, to obtain AuNPs with mean diameter of ca 32 nm. The size was assessed by using a Cary60 (Agilent, CA, USA) UV-Vis spectrophotometer confirming a 525 nm maximum LSPR wavelength.

Salt-induced aggregation test

In the wells of a microtitration plate, 250 μL of AuNPs at optical density of ca 1 were incubated with 25 μL of Ab solution corresponding to an Ab/OD ratio of 0-2-4-8-12-16-20-24-32-40 μg/OD. After 30 minutes of incubation at 37°C, 25 μL of a 10 % w/v NaCl in milliQ water was added to the wells. Then the plate was read using a bench photometer acquiring the absorbances at 540 nm and at 620 nm. The ratio between the absorbances at 540 nm and 620 nm was plotted against the amount of added Ab.

Fabrication of the LFIA strip

The AFB1-OVA competitor and SpA were diluted in phosphate buffer 20mM pH 7.4 and were spotted onto nitrocellulose membranes at 1 μL/cm by means of a XYZ3050 platform (Biodot, Irvine, CA, USA) to form the test and control lines, respectively. The conjugate pad was pre-adsorbed with the storage buffer and dried at 60°C for 1 hour. Subsequently, it was dipped into the Ab_AuNP conjugate solution (as diluted in the storage buffer to reach the appropriate optical density, OD) until complete saturation. Then, it was dried at room temperature for 2h. The membranes were dried at 37 °C for 60 min under vacuum, layered with sample, conjugate and adsorbent pads, cut into strips (5 mm width) by means of a CM4000 guillotine (Biodot), and inserted into plastic cassettes (Kinbio, Shanghai, China) to

fabricate the ready-to-use LFA device. Cassettes were stored in the dark in plastic bags containing silica at room temperature until use.

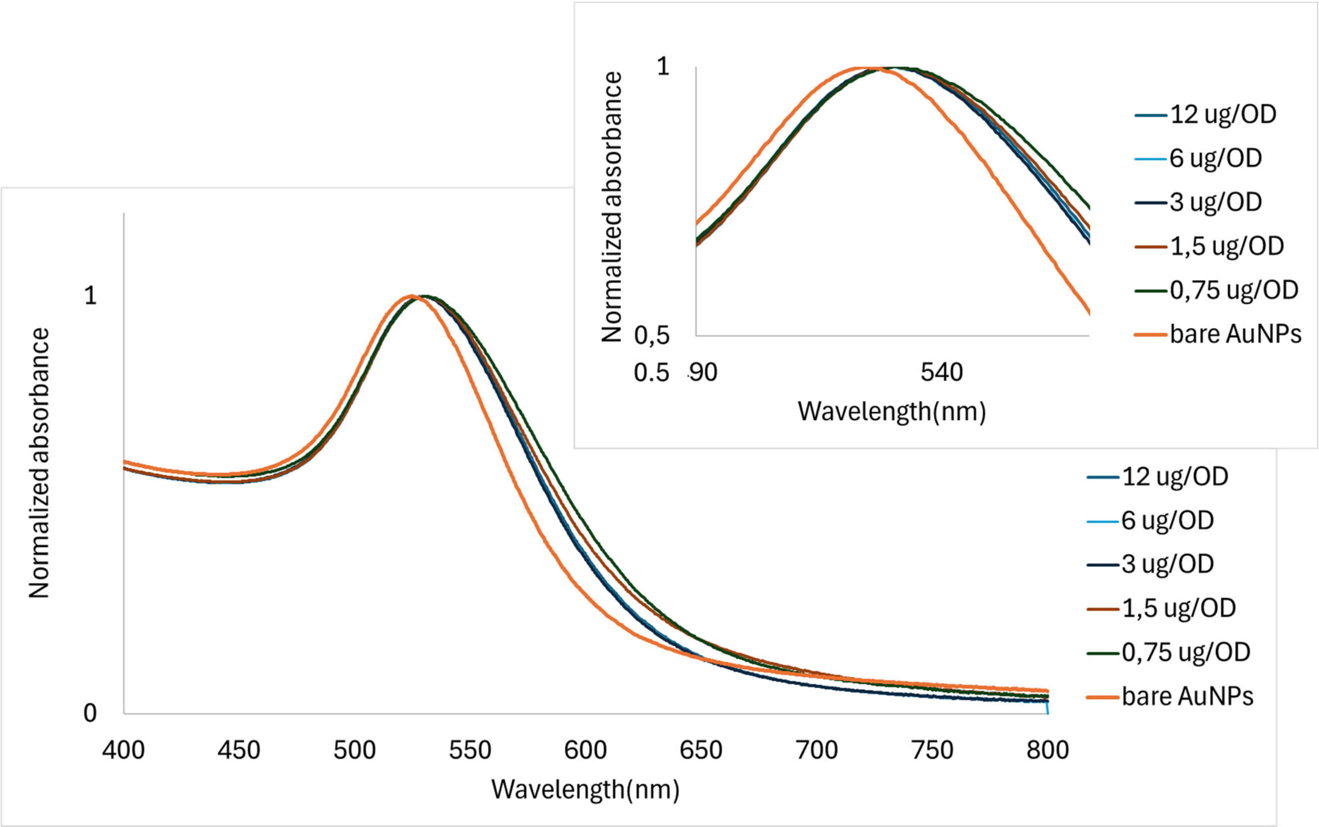

Figure S1. Visible spectra of the gold conjugates obtained by adsorbing different amounts of the anti-AFB1 antibody on AuNP that had optical density at the maximum of the LSPR band equal to 1. In the top right corner, the detail of the maximums of the LSPR of the different conjugates.

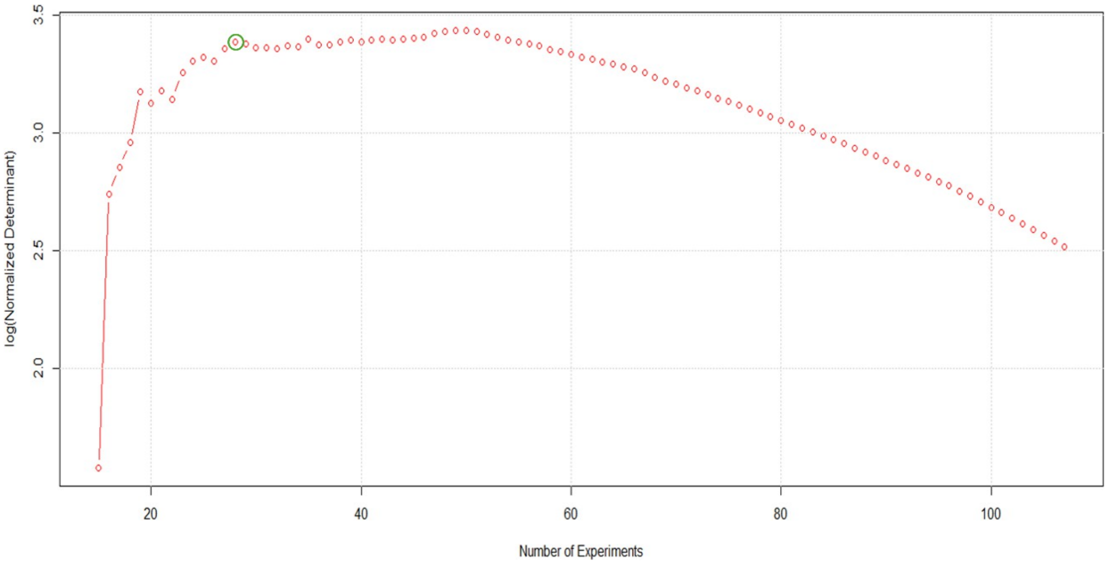

Figure S2. D-optimal evaluation of the number of experiments providing sufficient information for experimental space description.

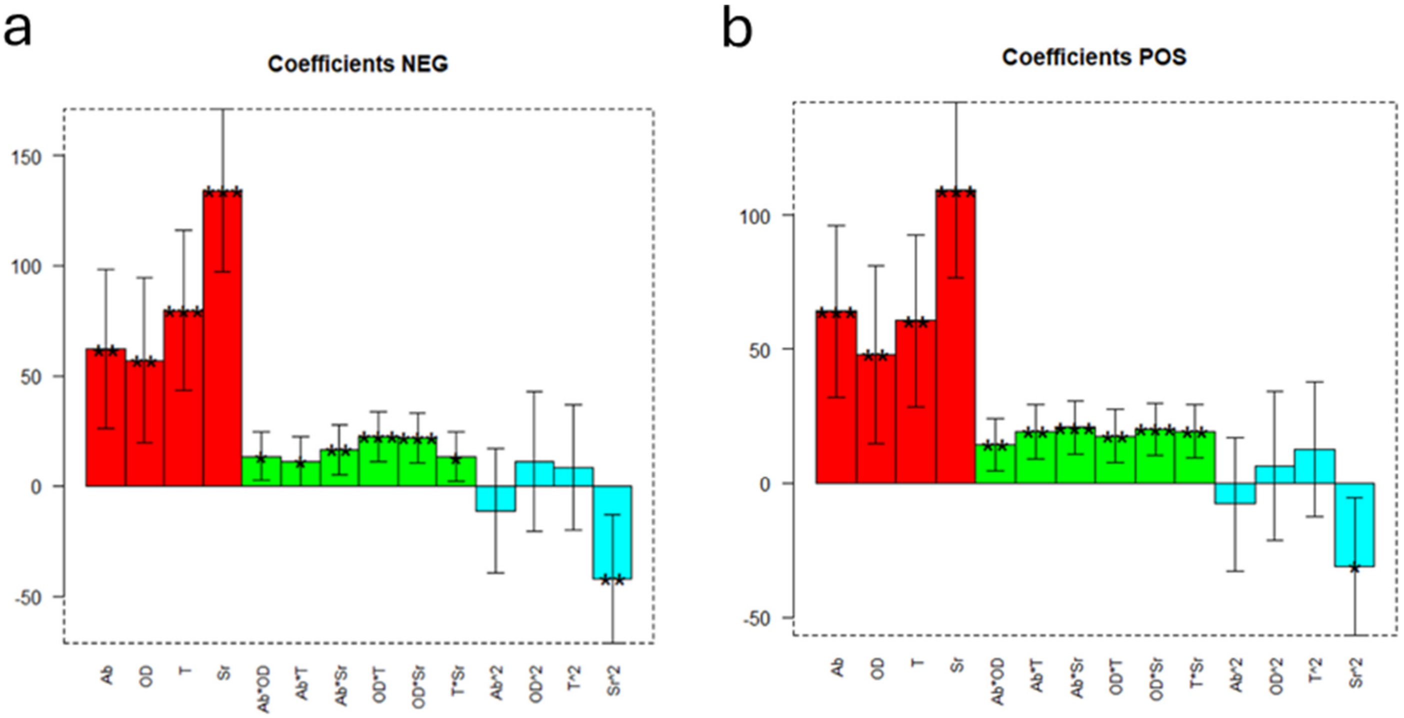

Figure S3. The coefficient plot of the NEG (a) and POS (b) models form the datasets. The significance was indicated as * = p < 0.05; ** = p < 0.05; and *** = p.< 0.001.

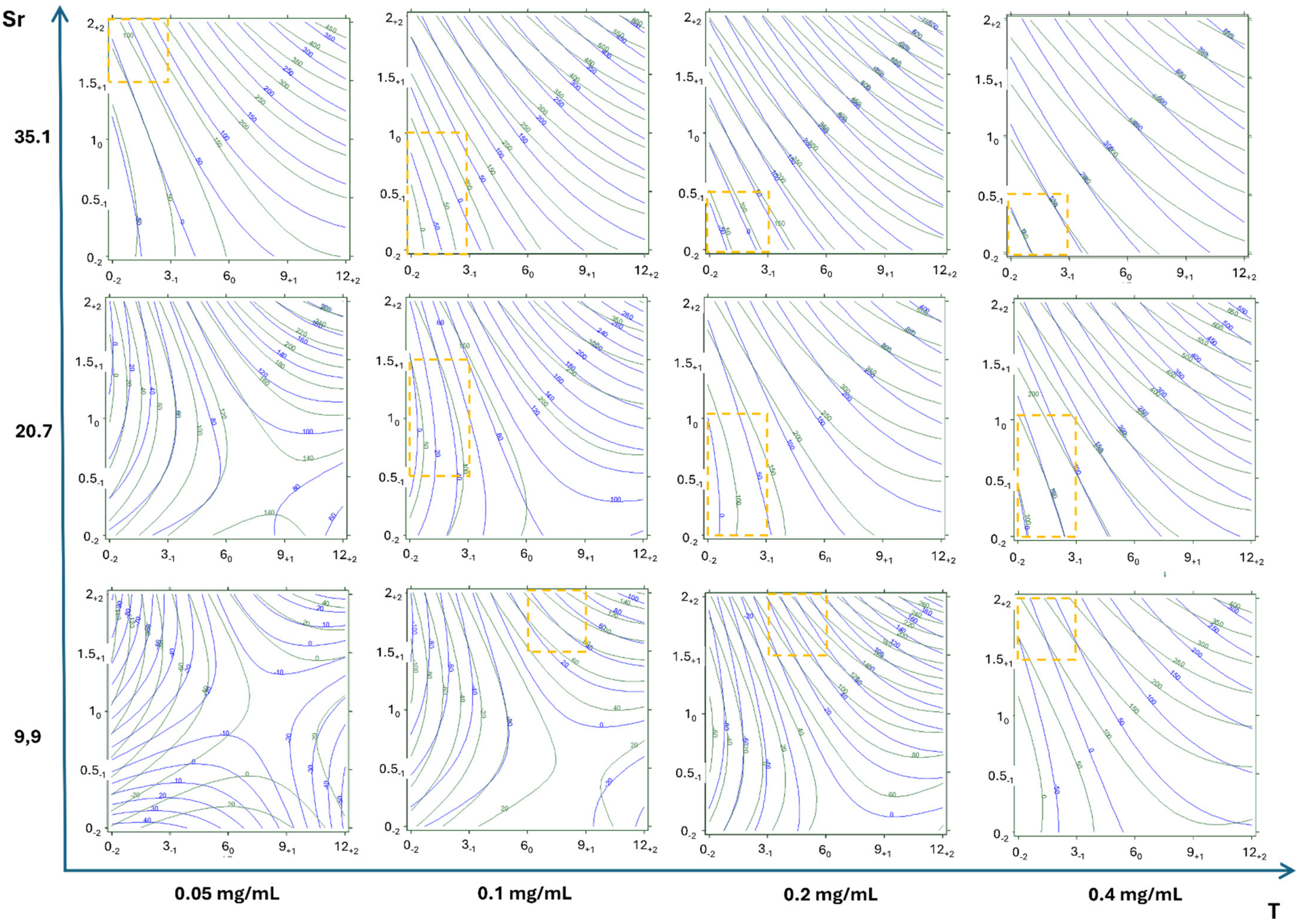

Figure S4. The overlay between NEG (blue) and POS (green) models from the START datasets. The x-axis represents antibody/OD ratios, expressed as μg/OD, and y-axis represents the optical density of the Ab_AuNP, expressed as arbitrary units. The corresponding levels of the DoE are indicated as subscripts. Yellow squares identify regions where NEG were above 80 a.u (true negative) and POS were below 15 a.u. (true positive).

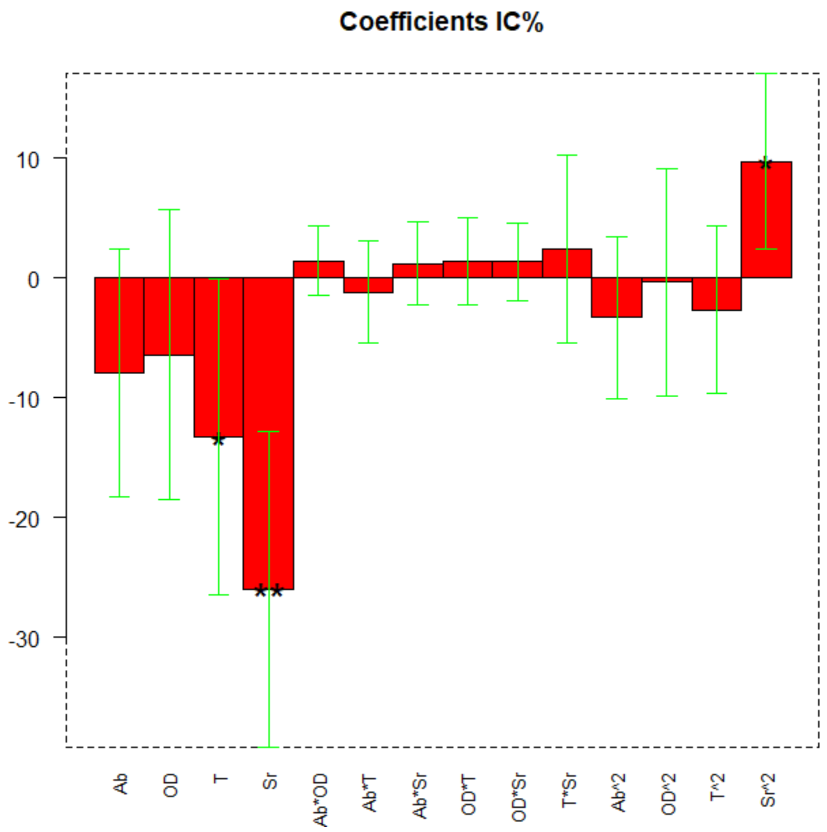

Figure S5. The coefficient plot of the IC% from the START datasets. The significance was indicated as * = p < 0.05; ** = p < 0.05; and *** = p < 0.001.

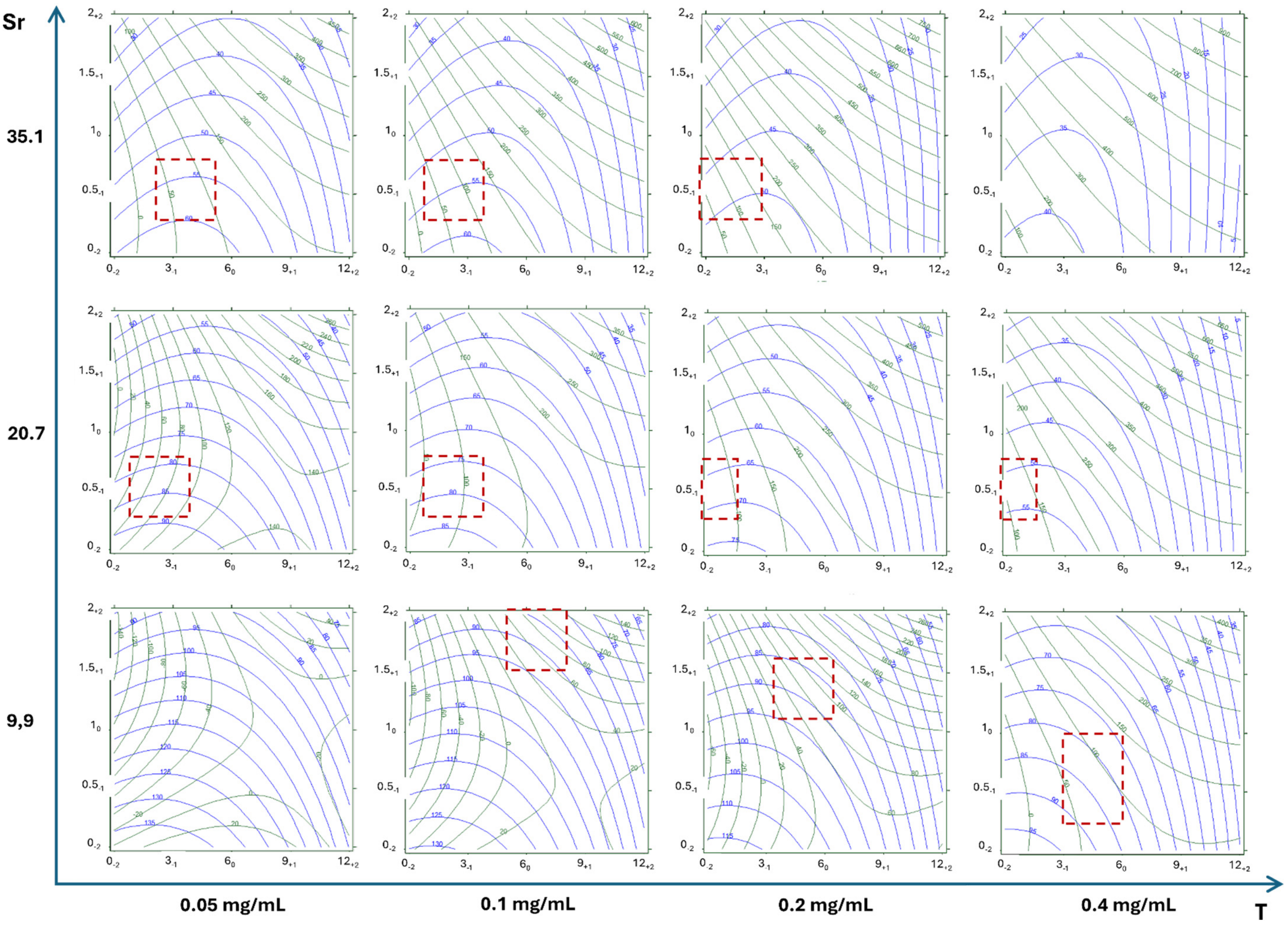

Figure S6. The overlay between NEG (blue) and IC% (green) models from the START datasets. The x-axis represents antibody/OD ratios, expressed as µg/OD and y-axis represents the optical density of the Ab_AuNP, expressed as arbitrary units. The corresponding levels are indicated as subscripts. Red squares identify regions where NEG were above 80 a.u (true negative) and POS were below 15 a.u. (true positive).

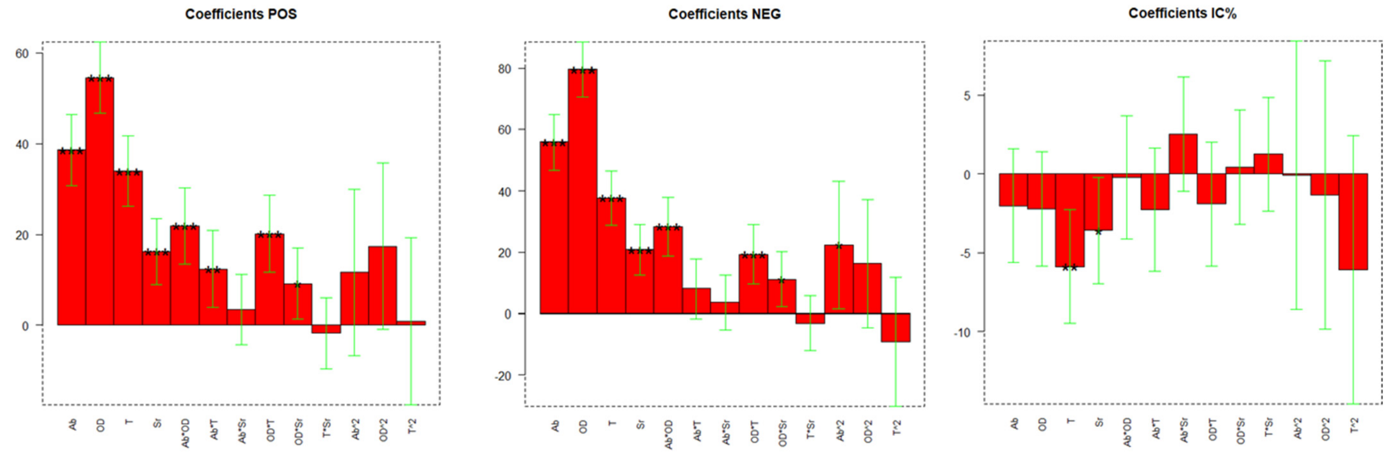

Figure S7: The coefficient plot of the NEG, POS, and IC% from the SHARPEN 1 dataset. The significance was indicated as * = p < 0.05; ** = p < 0.05; and *** = p < 0.001.

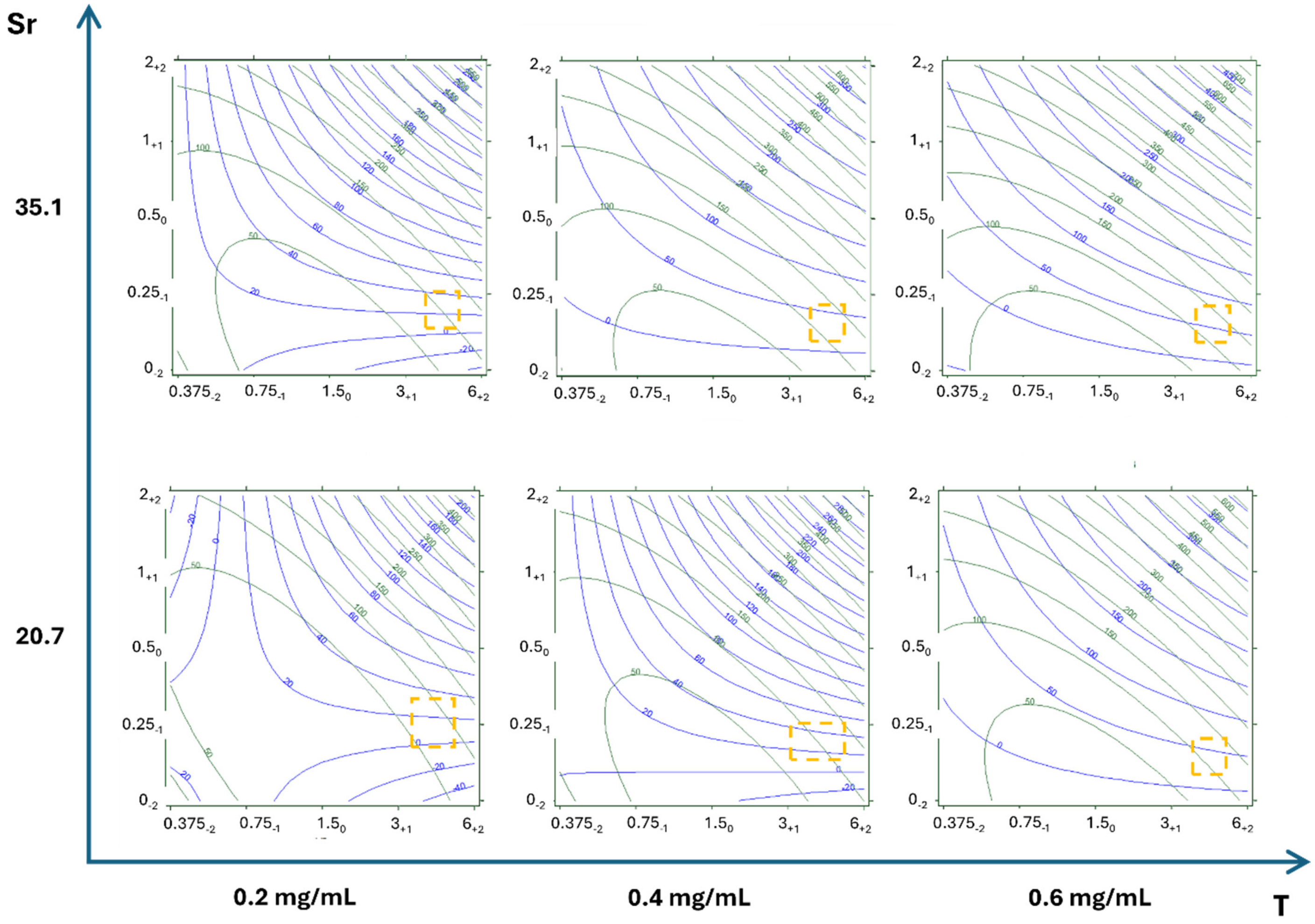

Figure S8. The overlay between NEG (blue) and POS (green) models from the START datasets. The x-axis represents antibody/OD ratios, expressed as μg/OD and y-axis represents the optical density of the Ab_AuNP, expressed as arbitrary units. The corresponding levels are indicated as subscripts. Yellow squares identify regions where NEG were above 80 a.u (true negative) and POS were below 15 a.u. (true positive).

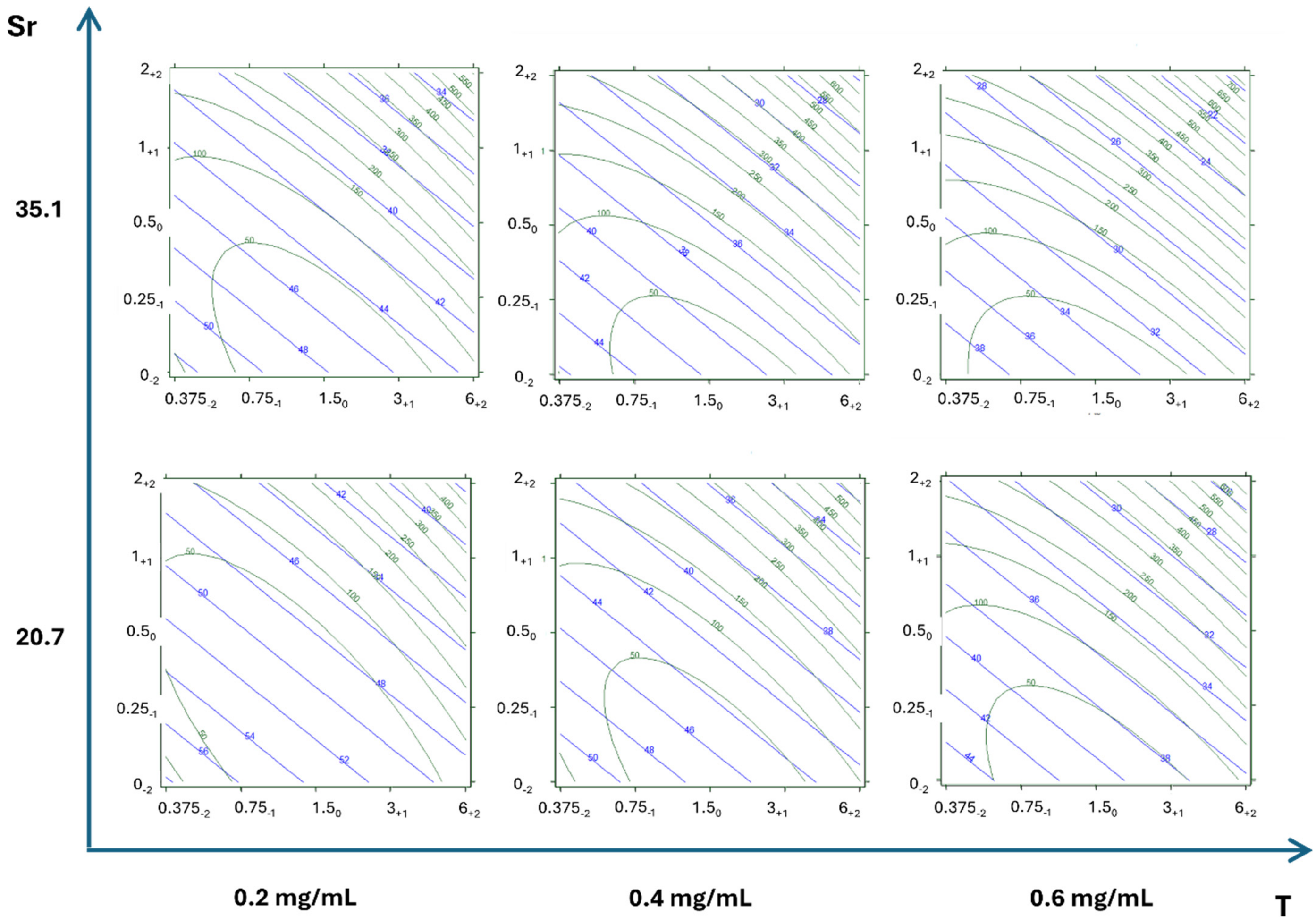

Figure S9. The overlay between NEG (blue) and IC% (green) models from the START datasets. The x-axis represents antibody/OD ratios, expressed as μg/OD and y-axis represents the optical density of the Ab_AuNP, expressed as arbitrary units. The corresponding levels are indicated as subscripts. No regions were found where NEG were above 80 a.u (true negative) and POS were below 15 a.u. (true positive).

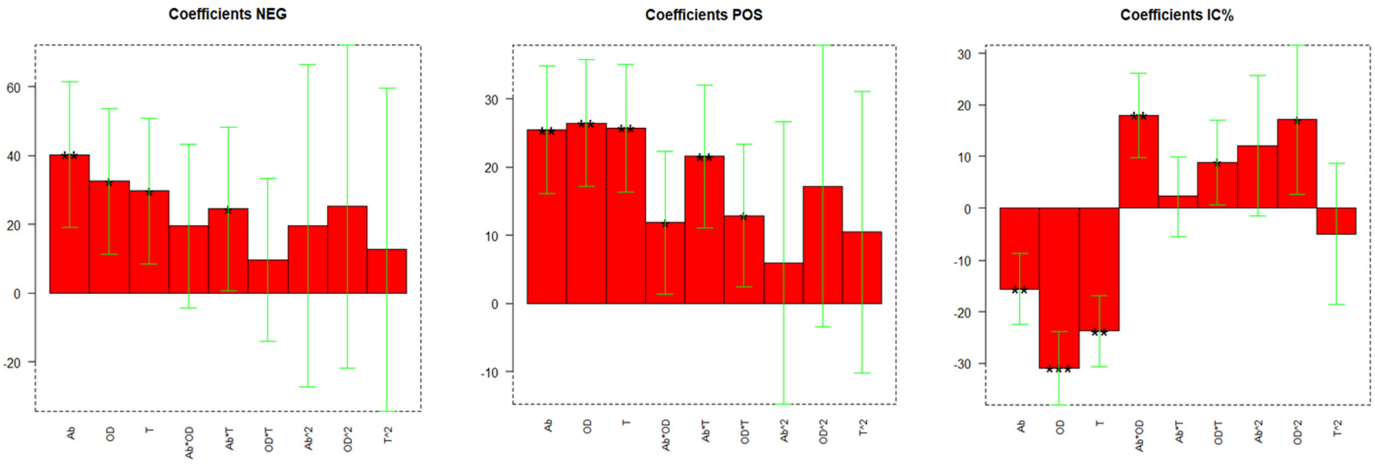

Figure S10. The coefficient plot of the NEG, POS, and IC% from the SHARPEN 2 datasets. The significance was indicated as * = $p < 0.05$; ** = $p < 0.05$; and *** = $p < 0.001$.

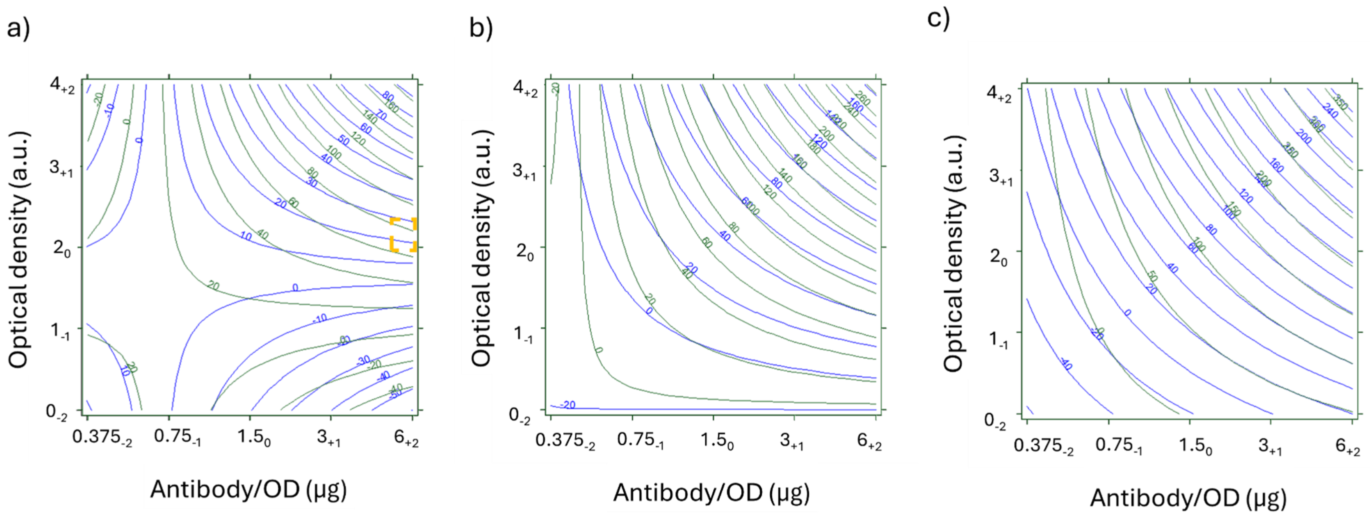

Figure S11. The overlay between NEG (blue) and POS (green) models from the SHARPEN 2 datasets. The Sr was fixed at 9.9 and T varied between 0.2 mg/L (a), 0.4 mg/L (b), and 0.6 mg/L (c) . The x-axis represents antibody/OD ratios, expressed as µg/OD and y-axis represents the optical density of the Ab_AuNP, expressed as arbitrary units. The corresponding levels are indicated as subscripts. Yellow squares identify regions where NEG were above 80 a.u (true negative) and POS were below 15 a.u. (true positive).

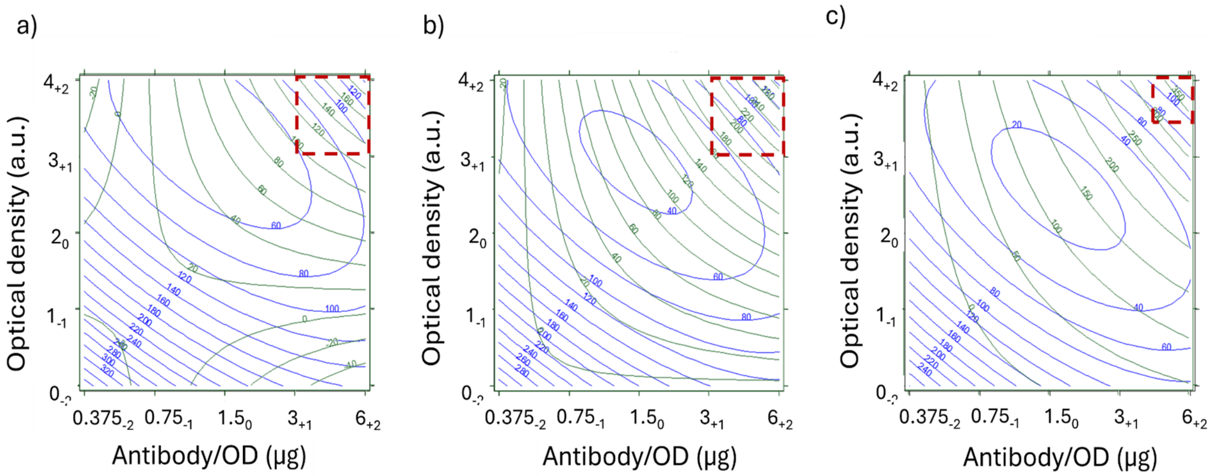

Figure S12. The overlay between NEG (blue) and IC% (green) models from the SHARPEN 2 datasets. The Sr was fixed at 9.9 and T varied between 0.2 mg/L (a), 0.4 mg/L (b), and 0.6 mg/L (c).. The x-axis represents antibody/OD ratios, expressed as µg/OD and y-axis represents the optical density of the Ab_AuNP, expressed as arbitrary units. The corresponding levels are indicated as subscripts. Red squares identify regions where NEG were above 80 a.u (true negative) and POS were below 15 a.u. (true positive).

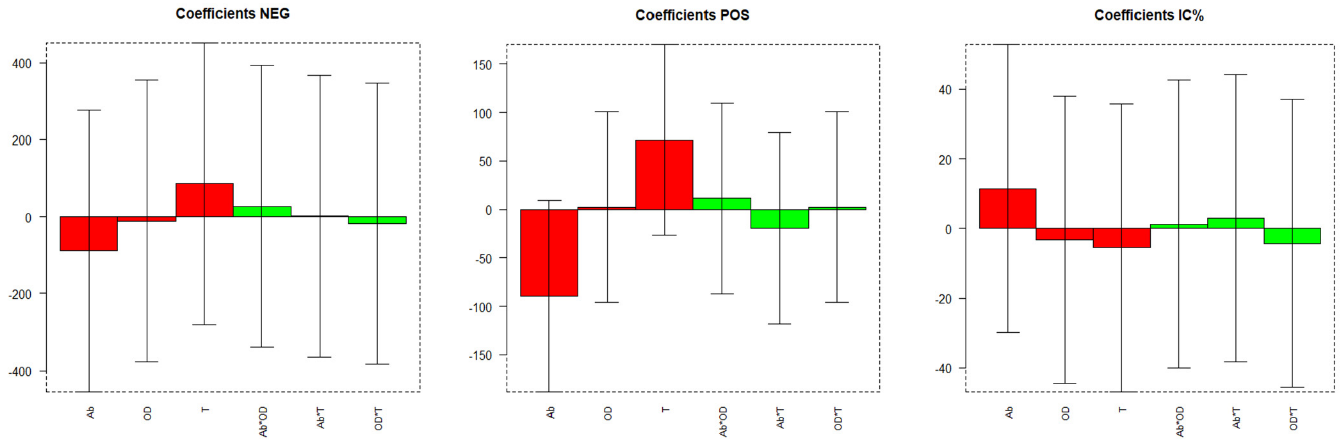

Figure S13. The coefficient plot of the NEG, POS, and IC% from the SHARPEN 3 datasets. The significance was indicated as * = $p < 0.05$; ** = $p < 0.05$; and *** = $p < 0.001$.

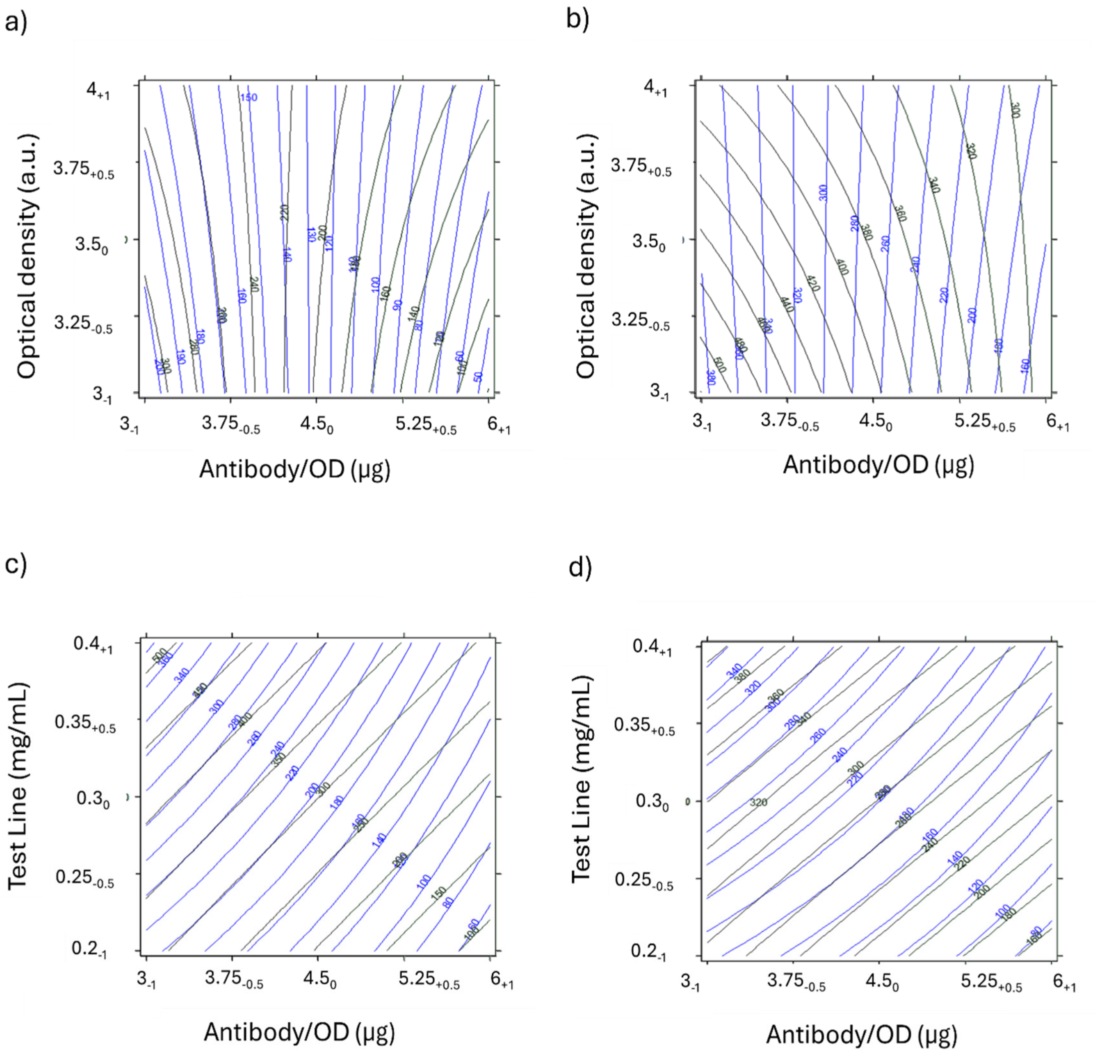

Figure S14. The overlay between NEG (blue) and POS (green) models from the SHARPEN 3 datasets. The Sr was fixed at 9.9. The plots represent optical density vs antibody/AuNP amounts for T=0.2 mg/mL (a) and 0.4 mg/mL (b) and test line concentration vs antibody/AuNP amounts for OD=3 (c) and 4.(d), respectively. Subscripts indicate the corresponding levels in the DoE. No regions were found where true positive and true negative results coexisted.

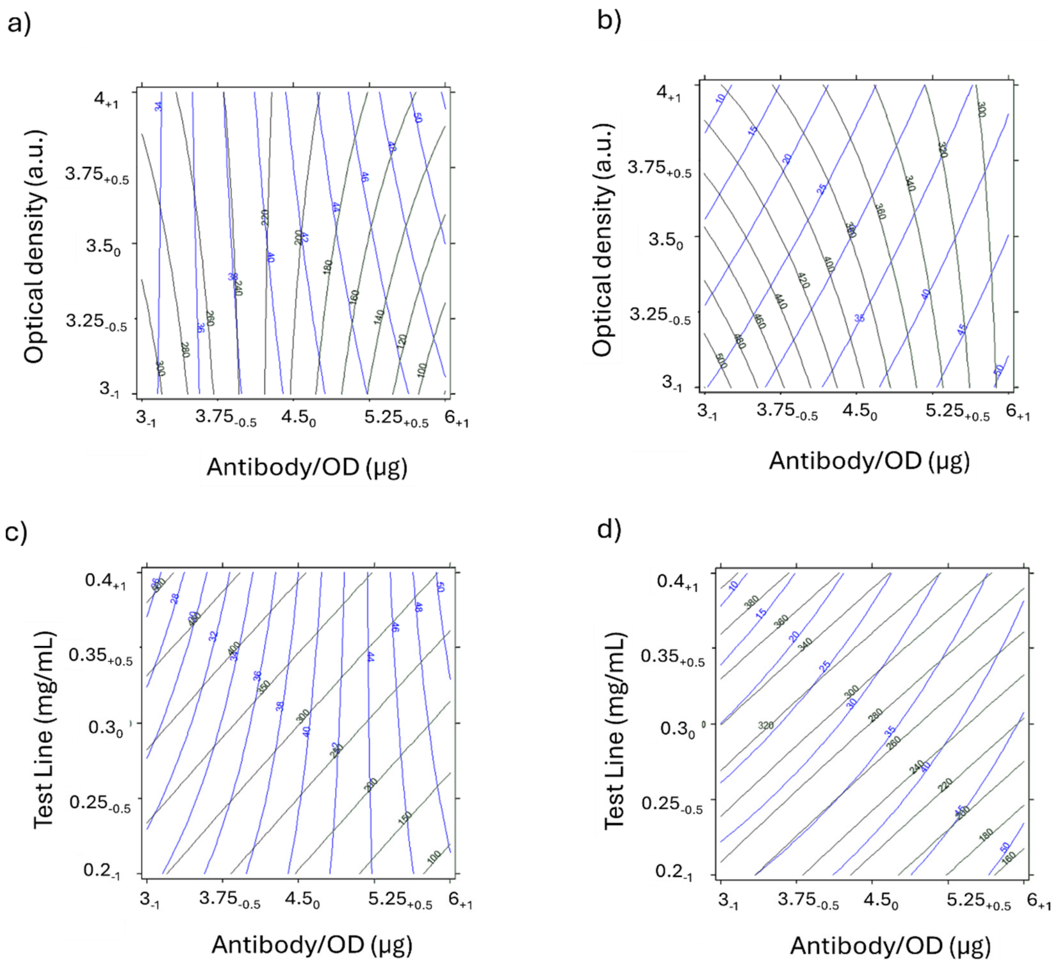

Figure S15. The overlay between NEG (blue) and IC% (green) models from the SHARPEN 3 datasets. The Sr was fixed at 9.9. The plots represent optical density vs antibody/AuNP amounts for T=0.2 mg/mL (a) and 0.4 mg/mL (b) and test line concentration vs antibody/AuNP amounts for OD=3 (c) and 4.(d), respectively. Subscripts indicate the corresponding levels in the DoE. No regions were found where true positive and true negative results coexisted.

Table S1. The coefficients of the multivariate model obtained from the Start, sharpen 1, sharpen2, sharpen 3 datasets including the significant classes of coefficients.

	**START**			**SHARPEN 1**			**SHARPEN 2**			**SHARPEN 3**		
DoE												
	NEG	POS	IC%	NEG	POS	IC%	NEG	POS	IC%	NEG	POS	IC%
b0	248.049	159.016	61.376	112.385	89.093	-38.819	63.13	33.91	-49.196	290.25	198.00	36.00
x Ab	62.284	64.150	-3.967	55.816	38.648	-2.292	39.21	24.71	-15.582	-89.00	-89.50	11.50
x OD	57.189	48.063	-2.939	79.559	54.570	-2.833	33.65	27.01	-30.978	-11.25	2.25	-3.25
x T	79.793	60.611	-11.109	37.688	33.970	-6.375	28.52	25.06	23.772	86.00	71.50	-5.50
x Sr	133.961	109.489	-21.981	20.730	15.290	-2.752	n.c.[a]	n.c.[a]	n.c.[a]	n.c.[a]	n.c.[a]	n.c.[a]
x AbxOD	13.600	14.441	n.c.[a]	28.346	21.708	n.c.[a]	19.84	12.36	17.985	27.50	11.25	1.25
x AbxT	11.316	19.279	n.c.[a]	8.046	12.208	n.c.[a]	22.84	20.32	2.248	1.75	-19.50	3.00

x AbxSr	16.797	20.835	n.c.[a]	3.541	3.423	n.c.[a]	n.c.[a]	n.c.[a]	n.c.[a]	n.c.[a]	n.c.[a]	n.c.[a]
x ODxT	22.622	17.666	n.c.[a]	19.246	19.958	n.c.[a]	10.70	12.87	8.890	-18.00	2.25	-4.25
x ODxSr	22.003	20.158	n.c.[a]	11.084	9.145	n.c.[a]	n.c.[a]	n.c.[a]	n.c.[a]	n.c.[a]	n.c.[a]	n.c.[a]
x TxSr	13.295	19.383	n.c.[a]	-3.188	-1.755	n.c.[a]	n.c.[a]	n.c.[a]	n.c.[a]	n.c.[a]	n.c.[a]	n.c.[a]
x Ab^2	-11.142	-7.690	-4.836	22.293	n.c.[a]	n.c.[a]	n.c.[a]	n.c.[a]	12.065	n.c.[a]	n.c.[a]	n.c.[a]
x OD^2	11.119	6.509	-1.831	16.293	n.c.[a]	n.c.[a]	n.c.[a]	n.c.[a]	17.134	n.c.[a]	n.c.[a]	n.c.[a]
x T^2	8.596	12.421	-4.297	-9.207	n.c.[a]	n.c.[a]	n.c.[a]	n.c.[a]	-4.983	n.c.[a]	n.c.[a]	n.c.[a]

[a.] n.c.: not considered in the model. [b.] b: explained variance %.

Table S2. START dataset including the color intensities acquired from the test line. The IC% were calculated as (NEG-POS)/NEG*100.

NEG	POS	IC%	Ab	OD	T	Sr
792	636	20	0	2	1	2
570	401	30	-1	2	1	2
651	592	9	2	0	1	2
425	426	0	2	-1	1	2
260	141	46	-1	-1	1	2
783	676	14	2	2	0	2
201	113	44	-1	-1	0	2
490	404	18	2	2	-2	2
190	118	38	-1	2	-2	2
106	63	41	-1	0	-2	2
237	146	39	2	-1	-2	2
105	44	58	0	-1	-2	2
741	666	10	2	2	1	0
285	171	40	0	-1	1	0
333	183	45	-1	2	0	0
155	87	44	2	0	-2	0
0	0	n.c.[a]	-1	-1	-2	0
434	302	30	2	2	1	-1
163	70	57	-1	2	1	-1
155	90	42	2	-1	1	-1
60	6	90	-1	-1	1	-1
37	0	100	0	0	0	-1
54	20	63	2	-1	0	-1
0	0	n.c.[a]	2	2	-2	-1
0	0	n.c.[a]	0	2	-2	-1
0	0	n.c.[a]	-1	2	-2	-1
0	0	n.c.[a]	2	-1	-2	-1
0	0	n.c.[a]	-1	-1	-2	-1

[a.] n.c.: not calculated for no starting signal.

Table S3. SHARPEN 1 dataset including the color intensities acquired from the test line. The IC% were calculated as (NEG-POS)/NEG*100.

NEG	POS	IC%	Ab	OD	T	Sr

399	307	23	1	1	1	1
210	137	35	-1	1	1	1
100	72	28	-1	0	1	1
114	76	34	1	-1	1	1
93	63	32	0	-1	1	1
52	35	33	-1	-1	1	1
194	115	41	-1	1	0	1
123	79	36	0	0	0	1
99	59	41	1	-1	0	1
276	173	37	1	1	-1	1
166	99	40	0	1	-1	1
112	74	34	-1	1	-1	1
201	113	44	1	0	-1	1
70	37	47	1	-1	-1	1
29	19	36	-1	-1	-1	1
359	272	24	1	1	1	-1
208	162	22	0	1	1	-1
166	109	34	-1	1	1	-1
186	111	41	1	0	1	-1
118	88	25	1	-1	1	-1
42	23	46	-1	-1	1	-1
295	191	35	1	1	0	-1
26	10	63	-1	-1	0	-1
181	88	51	1	1	-1	-1
63	30	53	-1	1	-1	-1
25	13	48	-1	0	-1	-1
43	28	36	1	-1	-1	-1
21	8	62	0	-1	-1	-1
14	8	42	-1	-1	-1	-1

Table S4. SHARPEN 2 dataset including the color intensities acquired from the test line. The IC% were calculated as (NEG-POS)/NEG*100.

NEG	POS	IC%	Ab	OD	T
249	173	31	1	1	1
55	43	23	-1	1	1
50	38	23	0	0	1
99	57	42	1	-1	1
28	0	100	-1	-1	1
34	18	47	-1	1	0
65	39	41	1	0	0
25	0	100	0	-1	0
98	36	63	1	1	-1
45	27	40	0	1	-1
36	16	56	-1	1	-1
28	0	100	-1	0	-1
30	0	100	1	-1	-1
0	0	n.c.[a]	-1	-1	-1

Table S5. SHARPEN 3 dataset including the color intensities acquired from the test line. The IC% were calculated as (NEG-POS)/NEG*100.

NEG	POS	IC%	Ab	OD	T
108	53	51	1	-1	-1
262	137	48	1	-1	1
119	60	49	1	1	-1
316	184	42	1	1	1
287	200	30	-1	-1	-1
549	393	28	-1	-1	1
303	193	36	-1	1	-1
378	364	4	-1	1	1
